# Symptom Network Dynamics during Antipsychotic Treatment in First-Episode Psychosis

**DOI:** 10.1093/schbul/sbag016

**Published:** 2026-03-21

**Authors:** Melissa G Zandstra, Floortje E Scheepers, Gabriela Lunansky, Silvana Galderisi, Birte Y Glenthøj, Inge Winter-van Rossum, Metten Somers, Edwin van Dellen

**Affiliations:** Department of Psychiatry, University Medical Center Utrecht, Utrecht, 3584 CX, The Netherlands; Department of Psychiatry, University Medical Center Utrecht, Utrecht, 3584 CX, The Netherlands; Department of Epidemiology and Data Science, Amsterdam University Medical Centers, VUMC, Amsterdam, 1081 BT, The Netherlands; Department of Mental and Physical Health and Preventive Medicine, University of Campania, Luigi Vanvitelli, Naples, 81100, Italy; Center for Neuropsychiatric Schizophrenia Research (CNSR) and Center for Clinical Intervention and Neuropsychiatric Schizophrenia Research (CINS), Copenhagen University Hospital, Mental Health Center Glostrup, Glostrup, 2600, Denmark; Department of Clinical Medicine, Faculty of Health and Medical Sciences, University of Copenhagen, Copenhagen, 2200, Denmark; Department of Psychiatry, University Medical Center Utrecht, Utrecht, 3584 CX, The Netherlands; Department of Psychiatry, Icahn School of Medicine, Mount Sinai, New York, 10029, United States; Department of Psychiatry, University of Oxford, Warneford Hospital, Oxford, OX3 7JX, United Kingdom; Department of Psychiatry, University Medical Center Utrecht, Utrecht, 3584 CX, The Netherlands; Department of Psychiatry, University Medical Center Utrecht, Utrecht, 3584 CX, The Netherlands; Department of Neurology, UZ Brussel and Vrije Universiteit Brussel, Brussels, 1090, Belgium

**Keywords:** Cross-Lagged Panel Network model, network intervention analysis, treatment response, amisulpride, olanzapine

## Abstract

*Background and Hypothesis:* Treatment response in first-episode psychosis varies substantially, yet underlying factors remain poorly understood. Symptom network theory suggests that inter-symptom relationships may influence treatment response. We hypothesized that symptom networks at baseline, as well as dynamic interactions over time, would differ between remitters and non-remitters, and that specific antipsychotics would show differential symptom-targeting effects.

*Study Design:* We compared baseline and temporal symptom networks between remitters (*n* = 250) and non-remitters (*n* = 196) from the OPTiMiSE trial using 21-item Positive and Negative Syndrome Scale (PANSS) data. Baseline networks were estimated using Gaussian graphical models and compared with the Network Comparison Test. Temporal networks across baseline, week 2, and week 4 were modeled using Cross-Lagged Panel Network analysis. Key symptoms were identified by in- and out-prediction values. Group differences were assessed via non-zero edge weight correlations and Jaccard Index (JI). Network Intervention Analysis was used to examine differential effects of continuing amisulpride versus switching to olanzapine in non-responders (*n* = 85).

*Study Results:* Baseline networks did not differ between outcome groups. However, temporal networks showed substantial differences: remitters and non-remitters had minimal overlap in symptom connections (baseline→week 2: JI = 0.014; week 2 → week 4: JI = 0.055) and virtually no correlation in connection strengths (baseline→week 2: r = -0.089, *P* = .447; week 2 → week 4: r = 0.005, *P* = .968). Key nodes (highest in/out-prediction) differed between groups. No robust symptom-specific medication effects emerged.

*Conclusions:* Temporal symptom dynamics, rather than static baseline relationships, differentiate response trajectories and could inform future research on early markers of non-remission. Absence of antipsychotic-specific effects suggests generic treatment mechanisms.

## Introduction

Initial treatment for first-episode psychosis (FEP) fails to achieve remission in a substantial proportion of patients, contributing to an elevated suicide risk and long-term functional impairment.^1^^,^^2^ Despite decades of research, the factors underlying divergent treatment outcomes remain poorly understood. Traditional models cast psychotic disorders as latent diseases and evaluate efficacy by overall symptom reduction or diagnostic change. In practice, however, individual symptoms interact dynamically—delusional ideas often provoke social withdrawal, while hallucinations might fuel anxiety—and these interconnections may influence response trajectories.

Symptom network theory reframes mental disorders as systems of causally linked symptoms rather than passive indicators of an underlying pathology.^3^ Within this framework, autonomous symptoms activate one another in feedback loops that sustain and amplify the disorder. This approach has been discussed for its potential to optimize treatment planning and evaluation,^4^ and may also aid in understanding outcome variations. If symptoms form interconnected networks, treatment response might depend on network organization and its capacity for change. Comparing symptom network characteristics between treatment responders and non-responders could offer insights into why some patients achieve remission while others do not.

Research in depression has begun to demonstrate the clinical relevance of this network approach. Patients who respond to treatment show different baseline symptom network patterns than non-responders,^5–7^ suggesting prognostic value. However, psychosis research remains limited. Baseline centrality differences between treatment responders and non-responders have been described in patients with psychosis,^8^ but the single study specifically in FEP found no differences between outcome groups.^9^ However, this study constructed networks using composite domain scores from multiple questionnaires rather than individual symptom items, which may have obscured important symptom-level network differences. It is therefore unclear if baseline symptom network configurations can differentiate between eventual remitters and non-remitters in FEP.

Beyond baseline differences, it has been investigated how treatment affects symptom networks over time. A cross-sectional study with repeated measures demonstrated that antipsychotic treatment reduces overall network strength (ie, symptoms become less strongly connected to each other).^10^ Crucially, other work demonstrates that remitters' networks display greater flexibility in network structure and nodal strength compared to non-remitters.^9^ These findings highlight the potential of longitudinal network analysis to differentiate varying treatment responses.

However, these longitudinal approaches do not model how symptoms influence each other over time. Temporal network analysis addresses this limitation by modeling directed relationships between symptoms across time points, testing whether changes in 1 symptom predict subsequent changes in other symptoms. Zhang et al.^11^ used Cross-Lagged Panel Network (CLPN) analysis in FEP patients over 6 months, finding certain negative symptoms preceded changes in other symptoms. While providing insights into symptom dynamics, the relatively long measurement intervals make it difficult to isolate medication effects, and analyzing the total sample together obscures potential differences in temporal symptom dynamics between remitters and non-remitters. Understanding how temporal symptom interactions differ between these groups could provide important insights into treatment response or resistance.

Different treatments, particularly those with distinct pharmacological profiles, may influence symptom networks uniquely. Previous research has addressed this by examining medication-specific effects on symptom centrality through the construction of separate networks for different antipsychotics.^10^ Network intervention analysis (NIA) advances this research by embedding treatment directly into the network, allowing identification of which symptoms are directly targeted by specific medications.^12^ This enables more targeted treatment by focusing on medications that directly address patients' most prominent symptoms.

This study uses data from the Optimization of Treatment and Management of Schizophrenia in Europe (OPTiMiSE) trial^13^^,^^14^ to examine symptom networks before and during controlled antipsychotic treatment: a 4-week open-label amisulpride phase for all patients, followed by double-blind randomization of non-remitters to continue amisulpride or switch to olanzapine. We aimed to compare both baseline and temporal networks between remitters and non-remitters, and examine differential effects of continuing amisulpride versus switching to olanzapine on specific symptoms in initial non-remitters over the course of treatment.

## Methods

### Participants

In the OPTiMiSE trial, participants with FEP were recruited from 27 hospitals and psychiatric clinics across 14 European countries (Austria, Belgium, Bulgaria, Czech Republic, Denmark, France, Germany, Italy, the Netherlands, Poland, Romania, Spain, Switzerland, and the UK), as well as Israel. Eligible individuals were aged 18-40 and met the criteria for schizophrenia, schizophreniform disorder, or schizoaffective disorder according to the Diagnostic and Statistical Manual of Mental Disorders, 4th edition (DSM-IV). Diagnoses were verified using the Mini-International Neuropsychiatric Interview-Plus.^15^

Exclusion criteria were as follows: (1) more than 2 years between the onset of psychosis and study enrollment; (2) prior antipsychotic use exceeding 2 weeks in the previous year or a lifetime total of 6 weeks; (3) known intolerance to the medications used in the trial; (4) any contraindications for the study medications; (5) being under compulsory treatment, legal guardianship, or custody; and (6) pregnancy or breastfeeding during the trial.

For the current study, data from phases I and II of the original study were utilized. In phase I, participants received open-label treatment with amisulpride (200-800 mg per day) for 4 weeks. Out of the 481 participants who were assessed and provided informed consent, 446 were enrolled in this phase. Those who did not achieve symptomatic remission after the initial 4 weeks proceeded to phase II, a 6-week randomized, double-blind trial, in which participants either continued amisulpride (200-800 mg) or switched to olanzapine (5-20 mg). The transition from phase I to phase II occurred between day 1 and day 7 of phase II, depending on the dosage received in phase I. A total of 92 participants were enrolled in phase II. The study adhered to the ethical principles of the Declaration of Helsinki, with ethics approval obtained in all participating countries. All participants were fully informed about the study and provided written consent prior to participating.

### Measures

The Positive and Negative Syndrome Scale (PANSS)^16^ was used to characterize psychotic symptoms. The PANSS exists of 30 items, of which 7 measure positive symptoms, 7 negative symptoms, and 16 general symptoms. It has a minimum score of 30 and maximum score of 210. Symptomatic remission was measured according to the Andreasen criteria^17^: 8 specific symptoms (items P1, P2, P3, N1, N4, N6, G5, and G9) are at most only mildly present (maximum rating of “3”). In our study, we utilized a recently proposed 21-item version of the PANSS.^18^ This version excludes 9 items that showed low stability in network analyses. Stability here refers to how consistently items cluster with the same symptom group when the analysis is repeated on multiple bootstrap samples. In the development of the PANSS-21, Dal Santo et al. applied a conservative stability cutoff of 0.75, such that items appearing in their original symptom community in fewer than 75% of resampled networks were excluded, informed by Monte Carlo simulation guidelines indicating reduced stability below approximately 0.65–0.75.^19^ We used this 21-item version for 3 reasons. First, as demonstrated by Dal Santo et al., restricting analyses to structurally stable items is essential for network modeling, as items with low stability undermine the reliability of estimated symptom relationships. Second, focusing on stable items yields a more interpretable and reproducible network structure, as unstable items that shift between symptom dimensions obscure the meaning of network edges. Third, the reduced number of nodes improves the participant-to-variable ratio, enhancing the robustness of network estimation given the available sample size. The remaining 21 items are distributed across 4 symptom domains: Positive, Cognitive/Disorganized, Excited/Aggressive, and Negative (see Table S1 for details). PANSS assessments were conducted at baseline, week 1, 2, and 4 during phase I, and at week 6, 8, and 10 during phase II.

### Network Analysis

#### Cross-Sectional Networks

We estimated regularized partial correlation networks to compare the symptom structures of remitters and non-remitters at baseline. These networks consist of nodes (representing individual PANSS items) and edges (representing the statistical associations between symptoms). Due to the ordinal nature of the PANSS items, we constructed the networks using Spearman correlations.

To obtain sparse networks with reduced false-positive connections, we applied the Least Absolute Shrinkage and Selection Operator (LASSO) regularization technique.^20^ This approach implements an ℓ1 penalty that shrinks small edges toward zero, resulting in a more conservative network estimation. The degree of penalization was controlled by the hyperparameter γ, set to the default value of 0.5.^21^

To enable a direct visual comparison of the remitter and non-remitter networks, the node layout was standardized using the "averageLayout" function, ensuring consistent positioning. The maximum edge value was capped at 0.54 (based on the strongest edge of the 2 networks), ensuring that edge weights were comparable across networks.

Empirical comparisons of symptom networks may be influenced by differences in symptom severity and sample size.^5^ Therefore, to ensure that group-level differences were not driven by baseline severity scores and to derive equally sized groups, we performed a sensitivity analysis comparing networks of subgroups matched on baseline severity scores. Matching was conducted using the 'nearest neighbor' method.^22^

##### Network Centrality

To examine node centrality within the network, we followed prior research in psychosis and estimated both strength and one-step expected influence (EI) centrality.^11^^,^^23^ Node strength is defined here as the sum of the absolute values of all edge weights and provides a measure of overall connectivity irrespective of edge sign. Expected influence extends this measure by accounting for both the direction and magnitude of connections.^24^ Specifically, one-step EI is calculated as the sum of all edge weights connected to a given node, with positive edges increasing and negative edges decreasing the node's EI.

##### Network and Centrality Stability

In accordance with recommendations of Epskamp et al.,^25^ the robustness of the network model was assessed by estimating the accuracy of edge weights and the stability of centrality indices. To estimate the accuracy of the edge estimates, non-parametric bootstrapping with 1000 samples was done. The confidence intervals (CIs) obtained from these analyses can be used to determine the accuracy of edge weights, with narrower CIs indicating higher accuracy of estimated edges.^25^ We also evaluated the stability of the centrality indices by using a case-dropping bootstrap procedure. This approach examines whether centrality rankings remain consistent when portions of the data are randomly removed.^25^ For the EI and strength centrality, we calculated a correlation stability coefficient (CS-coefficient), which represents the maximum proportion of cases that can be dropped while maintaining a correlation of 0.70 or higher with the original centrality values (based on 1000 bootstrap samples). Following established guidelines, we used a CS-coefficient threshold of 0.25 to define network stability.^25^ Networks with CS-coefficients above this threshold were considered sufficiently stable for interpretation.

##### Comparison of Network Characteristics

To compare the remitter and non-remitter networks, we used the Network Comparison Test (NCT), which is a permutation test that assesses the difference between 2 networks.^26^ The NCT was performed with 1000 permutations to test for differences in both global strength (absolute sum of all edge weights), network structure (distribution of edge weights), and EI and strength centrality. When significant differences in network structure were detected, we performed post-hoc testing on individual edges using Holm-Bonferroni corrections for multiple comparisons.

All analyses were conducted using R statistical software version 4.3.2.^27^ For cross-sectional networks, we used bootnet (1.6)^25^ (estimateNetwork with EBICglasso) for estimation, qgraph (1.9.8)^21^ for visualization and centrality analysis (centralityPlot), and MatchIt (4.7.0)^22^ for matching. Stability was assessed with bootnet (using the same LASSO parameters as in the original estimation), and group differences were tested with NetworkComparisonTest (2.2.2).^28^

#### Temporal Networks

To model the temporal relationships between PANSS items during the first 4 weeks of treatment, we used the CLPN approach.^29^ This method captures directed relationships between symptoms across discrete time points, allowing us to examine how symptoms influence each other over the course of treatment. These directed paths represent the shared variation between a symptom at time t and another symptom (either the same or different) at time t + 1, while accounting for all other symptoms at time t. Although phase I consisted of measurements at baseline, week 1, week 2 and week 4, we only included baseline (T0), week 2 (T1) and week 4 (T2) in our analyses, as CLPN analysis requires equal time intervals between measurements.

To estimate the CLPN, we computed autoregressive and cross-lagged coefficients using regularized regressions with a penalized maximum likelihood and LASSO penalty,^20^ with penalty selection via 10-fold cross-validation (CV). While LASSO effectively identifies true zero paths, it biases non-zero edge weights toward zero^29^ and the CV approach often retains false positive connections.^30^ To address the bias in non-zero edge estimation, we implemented a two-step procedure following Rhemtulla et al.^29^ where paths estimated as zero in the first step were fixed to zero, and the remaining non-zero edges were re-estimated using standard, non-regularized regression within a structural equation modeling (SEM) framework. This approach maintains the sparse structure identified by LASSO while providing unbiased estimates for the non-zero paths.

The use of SEM also allowed us to examine whether the predictive relations across successive occasions were equal (eg, if the paths from T0 to T1 are equivalent to those from T1 to T2). We fitted two nested models: an unconstrained model that allows different values for paths across time points, and a constrained model where both the presence and values of paths from T0 to T1 are constrained to be equal to those from T1 to T2.^29^ Once both models have been fit to the data, they can be compared using a nested chi-square difference test and approximate fit measures (eg, Root Mean Square Error of Approximation Difference [RMSEAD]).^31^ If imposing constraints did not significantly worsen model fit, we selected the constrained model for parsimony. If the constraints significantly degraded model fit, we retained the unconstrained model to better capture temporal variations in symptom dynamics.

##### Network Centrality

To quantify symptom centrality in the temporal networks, two indices were calculated: in-prediction and out-prediction.^29^ In-prediction reflects the extent to which each symptom at a given timepoint is predicted by all other symptoms measured at the preceding timepoint. Out-prediction captures the extent to which each symptom predicts other symptoms at the subsequent timepoint. Both indices are expressed as proportions of explained variance (R^2^), ranging from 0 (no predictive value) to 1 (perfect prediction). Compared to traditional directed centrality measures, such as in-strength and out-strength—which quantify only the summed strength of directed associations—in-prediction and out-prediction explicitly capture the predictive relationships between symptoms across consecutive measurement points. As such, these prediction-based indices offer greater clinical insight by directly quantifying how strongly individual symptoms forecast and respond to changes in the broader symptom network over the course of treatment.

##### Network Stability

To assess network stability, we conducted bootstrap analyses (1000 iterations) using non-parametric bootstrap resampling. Bootstrap CIs were calculated for all edge weights to evaluate the precision and robustness of parameter estimates.

##### Comparison of Network Characteristics

Since the NCT has been developed specifically for cross-sectional data and has not been validated for longitudinal data analysis, we needed alternative methods to test differences between remitter and non-remitter temporal networks. Following previous CLPN literature, we used two complementary measures: the correlation between non-zero edge weights and the Jaccard Index (JI).^32–35^ Matrix correlations quantified the similarity of existing edge strengths, and the JI^36^^,^^37^ quantified overlap in edge presence (range 0-1). Details are provided in the Supplementary Materials.

For temporal networks, we applied glmnet (4.1.8)^38^ for regularized regressions, lavaan (0.6.17)^39^ for SEM to re-estimate the remaining non-zero edges, qgraph for visualization, and bootstrapLavaan (lavaan) for stability analysis.

### Network Intervention Analysis

For Phase II, we applied NIA^12^ to assess the differential effects of olanzapine and amisulpride at weeks 6, 8, and 10. A Mixed Graphical Model (MGM)^40^ including all PANSS items (continuous) and the treatment variable (binary) was used to estimate the symptom network. Since the networks were estimated on full datasets, we included all available data at each assessment point, which led to slight variations in sample sizes.

Consistent with our previous analyses, we estimated the network using LASSO regularization with CV to select the optimal tuning parameter. For the estimated network, we computed the predictability of each symptom, representing the proportion of variance explained by all the other symptoms in the network.^41^ In case of a differential effect of treatment, we performed non-parametric bootstrapping with 1000 samples.^25^ Besides the CIs of the estimated edge weights, the plot also shows the proportion of bootstrap samples that included the edge in the network. As the focus was on the differential effect of treatment, we concentrated only on links involving treatment.

Given that we were contrasting two active treatment conditions, any edge between the treatment variable and a specific symptom reflects a treatment-specific effect. Importantly, these direct effects highlight differences between the two treatments. If both treatments affect a symptom similarly, this effect will not be displayed in the network. Therefore, the absence of an edge should not be interpreted as the absence of a treatment effect but rather as the absence of a differential treatment effect between amisulpride and olanzapine.

We used the mgm package (version 1.2.14)^40^ for estimating the networks, the qgraph package for visualization, and the bootnet package for stability analyses.

## Results

### Sample Characteristics

The baseline sample of phase I included 446 participants, with 250 classified as remitters and 196 as non-remitters by the end of the phase. The mean age of the participants was 25.5 years (SD = 6.0), and 70% of the sample were male. A total of 386 participants completed the assessment at week 2, and 371 completed the final assessment. The baseline sample of phase II included 85 participants, with 43 randomized to receive amisulpride and 42 to receive olanzapine. Detailed baseline characteristics for both Phase I and Phase II are presented in Table 1. Sample characteristics for subsequent time points (Phase I: Table S2; Phase II: Table S3) and individual PANSS scores (Phase I: Table S4; Phase II: Table S5) are available in the Supplementary Materials.

**Table 1:** Baseline Sample Characteristics for Phase I and II

		**Phase I**			**Phase II**	
	**Full sample** **(*n* = 446)**	**Remitters** **(*n* = 250)**	**Non-remitters** **(*n* = 196)**	**Full sample** **(*n* = 85)**	**Amisulpride** **(*n* = 43)**	**Olanzapine** **(*n* = 42)**
Age, M (SD)	25.5 (6.0)	26.3 (6.3)	24.4 (5.3)	25.0 (5.5)	25.1 (5.5)	24.8 (5.6)
Sex, male, *N* (%)	312 (70.0%)	174 (69.6%)	138 (70.4%)	66 (77.6%)	35 (81.4%)	31 (73.8%)
Race, *N* (%) White Black Asian Other	386 (86.5%) 29 (6.5%) 17 (3.8%) 14 (3.1%)	215 (86.0%) 17 (6.8%) 10 (4.0%) 8 (3.2%)	171 (87.2%) 12 (6.1%) 7 (3.6%) 6 (3.1%)	79 (92.9%) 3 (3.5%) 1 (1.2%) 2 (2.4%)	40 (93.0%) 2 (4.7%) 0 1 (2.3%)	39 (92.9%) 1 (2.4%) 1 (2.4%) 1 (2.4%)
Disease type, *N* (%) Schizophrenia Schizoaffective Schizophreniform	229 (51.3%) 27 (6.1%) 190 (42.6%)	117 (46.8%) 19 (7.6%) 114 (45.6%)	112 (57.2%) 8 (4.1%) 76 (38.8%)	58 (68.2%) 1 (1.2%) 26 (30.6%)	31 (72.1%) 0 12 (27.9%)	27 (64.3%) 1 (2.4%) 14 (33.3%)
Duration of current episode, months, M (SD)	6.3 (6.2), *n* = 430	6.0 (6.0), *n* = 239	6.6 (6.6), *n* = 191	12.0 (2.7), *n* = 82	12.7 (2.8), *N* = 42	11.3 (2.4), *n* = 40
Education, years, M (SD)	12.3 (3.0), *n* = 437	12.5 (3.1), *n* = 246	12.0 (2.7), *n* = 191	8.4 (7.4), *n* = 83	9.8 (7.9), *n* = 42	7.0 (6.7), *n* = 41
PANSS total score^a^, M (SD)	54.7 (14.8)	51.5 (14.6)	58.9 (14.1)	52.4 (13.5)	53.7 (12.5)	51.2 (14.4)

Abbreviation: PANSS, Positive and Negative Syndrome Scale.

a Total score of the 21-item PANSS version used in this study, based on a previous network stability analysis study.^18^

### Cross-Sectional Networks

#### Comparison of Baseline Networks in Remitters and Nonremitters

**Figure 1:**
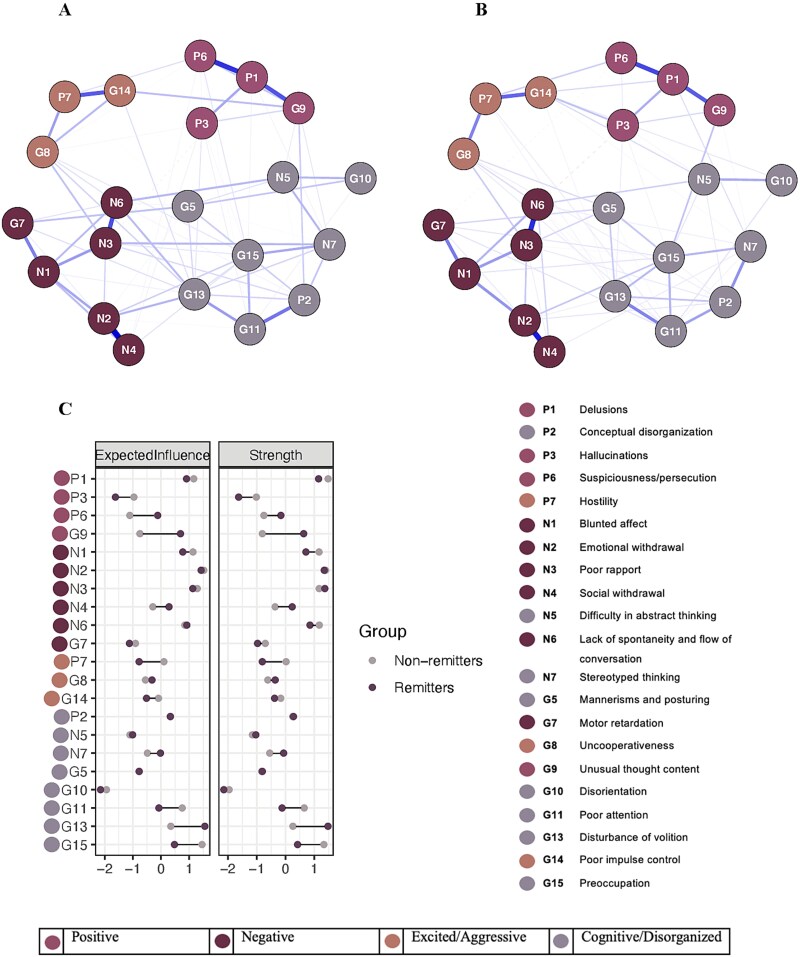
Baseline networks, expected influence (EI), and strength centrality for remitters and non-remitters. A, network of remitters (*n* = 250). B, network of non-remitters (*n* = 196). C, Z-scored EI and strength centrality for remitters and non-remitters. In the networks, blue solid lines represent positive associations, and red dashed lines represent negative associations. Edge thickness indicates the strength of the association, with thicker edges reflecting stronger connections; all edges were standardized to a maximum value of 0.54, corresponding to the strongest observed association.

Figure 1A and B show the baseline symptom networks for patients who responded to treatment by the end of Phase I (remitters) and those who did not (non-remitters). The remitter network contained 90 non-zero edges, while the non-remitter network contained 87 non-zero edges. There was no significant difference in the overall network structures between the two groups (M = 0.15, *P* = .94). Global strength also did not significantly differ between remitters (8.56) and non-remitters (7.94, S = 0.62, *P* = .48). Figure 1C shows the standardized EI and strength centrality for remitters and non-remitters. For remitters, disturbance of volition (G13) showed the highest EI and strength centrality, followed by emotional withdrawal (N2) and poor rapport (N3). In non-remitters, emotional withdrawal (N2) showed the highest EI, followed by preoccupation (G15) and poor rapport (N3), whereas strength centrality was highest for delusions (P1), followed by emotional withdrawal (N2) and preoccupation (G15). Despite these descriptive differences, no nodes showed statistically significant differences in EI or strength centrality between remitters and non-remitters. Sensitivity analyses using a matched sample yielded similar results, with no significant differences in overall network structure (M = 0.16, *P* = .79) or global strength (S = 0.57, *P* = .82) between remitters and non-remitters. Node-specific EI or strength values also did not differ significantly between groups. See Figure S1 for details. Bootstrapped 95% CIs of edge weights suggest that edges are fairly stable (Figure S2). EI and strength centrality estimates were also fairly stable (Figure S3), with a CS-coefficient of 0.44 for the remitters and 0.29 for the non-remitters. Complete edge weight matrices are provided in Tables S6 and S7.

**Figure 2:**
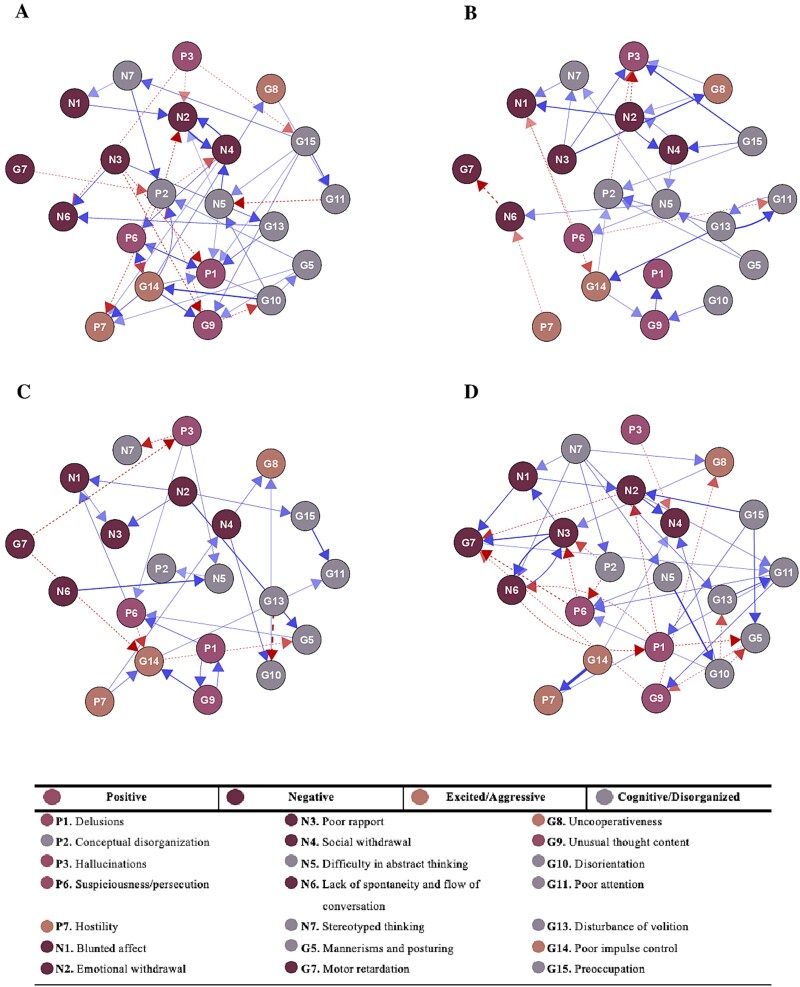
Cross-lagged panel network model plots for T0 → T1 network and T1 → T2 network of remitters and non-remitters. A, T0 → T1 remitters. B, T1 → T2 remitters. C, T0 → T1 non-remitters. D, T1 → T2 non-remitters. The direction of the edges indicates the direction of the cross-lagged coefficients between nodes. Dashed red edges signify negative associations, while blue edges represent positive associations. The saturation and thickness of the edges indicate the strength of the associations. All edges were standardized to a maximum value of 0.40, corresponding to the strongest observed association. Only significant (*P* < .05) edges are visualized.

### Temporal Symptom Dynamics

The primary analyses focused on comparing temporal symptom networks between remitters and non-remitters during antipsychotic treatment. For completeness, additional analyses of the full sample can be found in the Supplementary Materials.

#### Symptom Severity over Time

Item-level symptom severity over time for remitters and non-remitters showed distinct patterns (Figure S4, standard deviations in Table S4). Remitters showed substantial symptom improvement between T0 and T1, particularly for positive symptoms (P1 and P6), followed by a more gradual decline from T1 to T2**.**

In contrast, non-remitters exhibited a more heterogeneous and dynamic symptom trajectory. Between T0 and T1, most symptoms decreased moderately, but several symptoms increased in severity, including blunted affect (N1), lack of spontaneity and flow of conversation (N6), mannerisms and posturing (G5), and motor retardation (G7). From T1 to T2, although a few symptoms showed slight reductions, the majority showed renewed increases. These included delusions (P1), hostility (P7), blunted affect (N1), emotional withdrawal (N2), poor rapport (N3), social withdrawal (N4), lack of spontaneity and flow of conversation (N6), mannerisms and posturing (G5), motor retardation (G7), unusual thought content (G9), disturbance of volition (G13), and poor impulse control (G14). The most notable increase was observed in N6 (lack of spontaneity and flow of conversation), with the mean score rising from 2.79 at T1 to 3.07 at T2.

#### Testing Temporal Stability of Psychotic Symptom Relationships

As a first step, we examined whether symptom dynamics remained consistent across time intervals during antipsychotic treatment by testing the imposition of cross-time constraints. For remitters, the unconstrained model fitted the data well; χ^2^(df = 859) = 904.7, *P* = .136, RMSEA = .015 (.000,.024); CFI = .994, TLI = .988 (robust statistics reported). The constrained model showed acceptable fit; χ^2^ (df = 1091) = 1344.38, *P* < .001; RMSEA = .016 (.000,.025); CFI = .990, TLI = .984. The chi-square difference test showed a significant difference, χ^2^diff (df = 232) = 439.7, *P* < .001. This suggests that adding the cross-time constraints significantly reduces model fit, indicating that symptom dynamics changed significantly between the first-time interval (T0 to T1) and the second time interval (T1 to T2), a pattern also observed in non-remitters (χ^2^*diff* (*df* = 213) = 437.1, *P* < .001). Therefore, we used the unconstrained model for all subsequent analyses to accurately capture the temporal variations in symptom dynamics during antipsychotic treatment.

#### Comparison of Cross-Lagged Panel Networks in Remitters and Nonremitters

**Figure 3:**
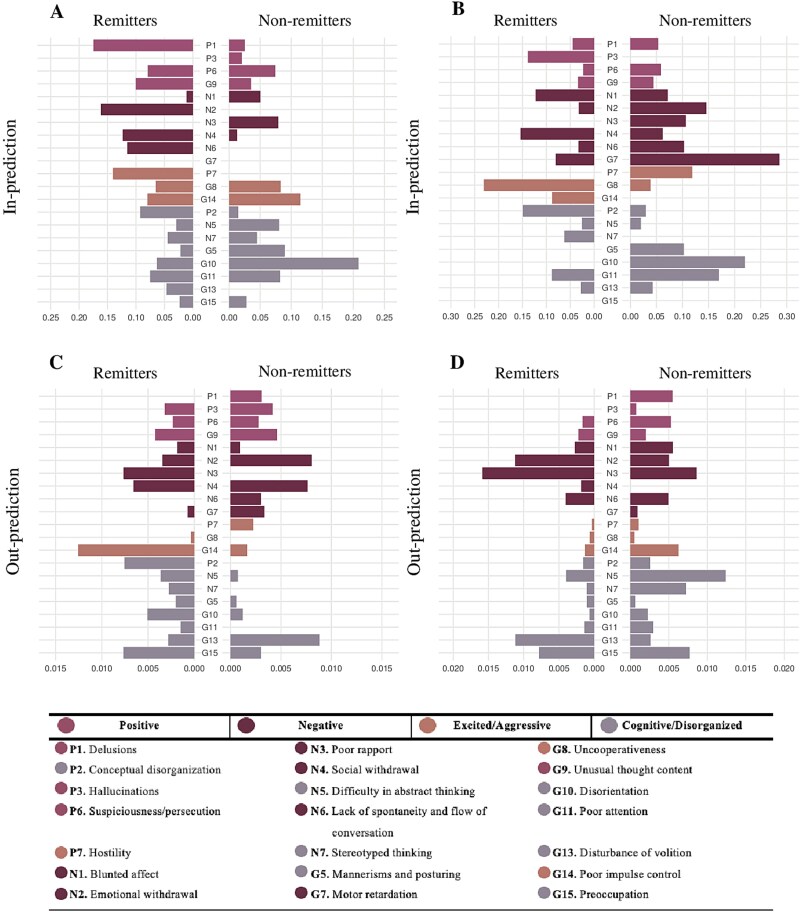
Cross-lagged in-and-out prediction plots for T0 → T1 and T1 → T2 for remitters and non-remitters. A, In-prediction plot T0 → T1. B, In-prediction plot for T1 → T2. C, out-prediction plot T0 → T1. D, out-prediction plot T1 → T2. The x-axis represents the explained variance.

Figures 2 and 3 present the directed symptom networks for remitters and non-remitters across both time intervals (T0 → T1 and T1 → T2), along with the in-and-out prediction values. The directed edges represent temporal associations between symptoms after controlling for all other symptoms at baseline (for T0 → T1) and week 2 (for T1 → T2). To enhance interpretability, autoregressive edges were excluded from the plots (see Figure S5 for complete networks including autoregressive edges). The mean weight of the autoregressive edges was 0.48 for the remitter T0 → T1 network, 0.51 for the non-remitter T0 → T1 network, 0.51 for the remitter T1 → T2 network, and 0.54 for the non-remitter T1 → T2 network (Figure S6).

**Figure 4:**
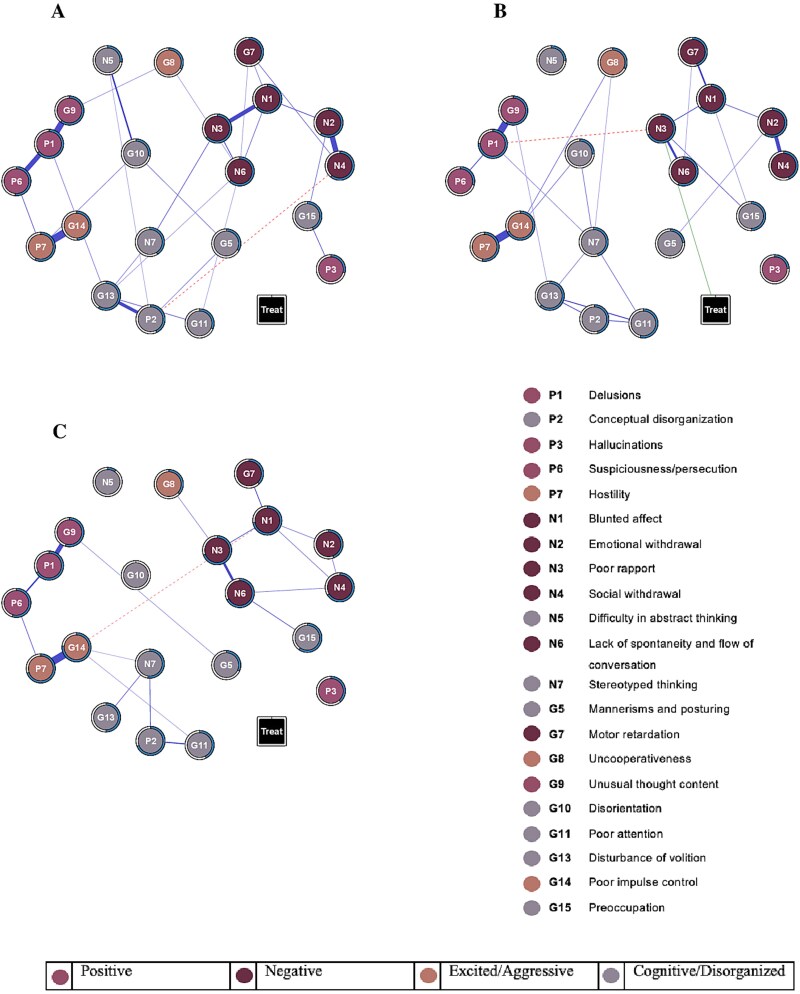
Network intervention analysis comparing amisulpride and olanzapine effects. A, week 6. B, week 8. C, week 10. The networks include the PANSS items (circles) and the treatment (square). Treatment includes amisulpride (scored as 0) and olanzapine (scored as 1). A green line indicates an effect in favor of olanzapine, and a yellow line indicates an effect in favor of amisulpride. For each node, the proportion of explained variance by all other nodes, that is, the predictability, is visualized by a ring around the node: A completely filled ring indicates that 100% of the variance is explained, a completely empty ring corresponds to an explained variance of 0%. Abbreviations: PANSS, Positive and Negative Syndrome Scale.

Network similarity analyses revealed substantial differences between remitters and non-remitters. There was minimal overlap in which symptom connections were present between groups (T0 → T1: JI = 0.014; T1 → T2: JI = 0.055, indicating that only 1.4% and 5.5% of all unique connections were shared), and the strength of non-zero edge weights showed virtually no correlation between groups (T0 → T1: *r* = -0.09, *P* = .45; T1 → T2: *r* = 0.005, *P* = .97). These findings indicate largely distinct patterns of symptom-to-symptom associations between remitters and non-remitters over time.

Examining these differences in more detail, remitters initially (T0 → T1) showed delusions (P1) as the most predicted symptom (*R^2^* = 0.18), influenced by a combination of positive (P6), negative (N3), excited/aggressive (G14) and cognitive/disorganized symptoms (N5, G13, G15). Additionally, the excited/aggressive symptom, poor impulse control (G14), emerged as the strongest predictor (*R^2^* = 0.012), influencing several positive (P1, P6, G9) and excited/aggressive (G8, P7) symptoms. These predictive patterns shifted in the T1 → T2 network, where the previously central nodes showed markedly lower in-or-out prediction values (P1: *R^2^* = 0.05; G14: *R^2^* = 0.001). Instead, the negative symptom poor rapport (N3) emerged as the strongest predictor (*R^2^* = 0.016), influencing cognitive/disorganized (N7), positive (P3), and excited/aggressive (G8) symptoms. Uncooperativeness (G8) became the most strongly predicted symptom (*R^2^* = 0.24), though its only predictor was N3.

For non-remitters, symptom dynamics followed a different trajectory. In the T0 → T1 network, the cognitive/disorganized symptom G10 (disorientation) was the most strongly predicted node (*R^2^* = 0.20), influenced by negative (N4) and cognitive/disorganized (G13) symptoms. G13 (disturbance of volition) was also the strongest predictor (*R^2^* = 0.006), although it only influenced G10. This shifted in the T1 → T2 network. The cognitive/disorganized symptom motor retardation (G7) emerged as the most predicted symptom (*R^2^* = 0.28), driven by negative (N1, N2, N3), positive (P6, G9) symptoms. At the same time, difficulty in abstract thinking (N5) became the strongest predictor (*R^2^* = 0.012), influencing P6 and G10. Though cross-lagged effects appear small, they remain meaningful given high symptom autocorrelations that constrain available variance for cross-symptom prediction.

Notably, in the non-remitter T1 → T2 network, all outgoing edges from positive symptoms were negative, indicating that lower levels of positive symptoms at week 2 were associated with higher levels of other symptoms at week 4, particularly in the negative and cognitive/disorganized domains. This pattern is reflected in the mean scores (Figure S4), which generally shows a continued decline in positive symptoms alongside increasing negative symptoms among non-remitters.

Complete edge weight matrices for all networks are provided in Tables S8-S11, detailing all directed associations between PANSS items for each group and time interval. Bootstrapped CIs around edge weights were moderate (see Figure S7).

### Differential Effects of Amisulpride and Olanzapine

Detailed item-level severity scores for both treatment groups across all assessment timepoints of phase II are presented in Table S5. Figure 4 shows the network models at week 6, week 8, and week 10. While there were no symptom-specific differences between treatments at weeks 6 and 10, olanzapine demonstrated a symptom-specific effect on poor rapport (N3) at week 8. However, stability analysis revealed that the link between treatment and poor rapport was retrieved in only 31% of the samples, indicating low stability (Figure S8).

## Discussion

This study provides novel insights into the symptom dynamics before and during antipsychotic treatment in FEP, comparing remitters and non-remitters and examining differential symptom-level effects of amisulpride versus olanzapine. Cross-sectional analyses revealed no significant differences between outcome groups. In contrast, temporal network analyses uncovered distinct patterns of symptom interactions at both intervals. Among remitters, early dynamics were characterized by delusions as the most strongly predicted symptom and poor impulse control as the dominant predictor; at the later interval, the emphasis shifted toward uncooperativeness and poor rapport. Non-remitters followed a different trajectory: disorientation was initially the most predicted symptom, and disturbance of volition the strongest predictor, later shifting to motor retardation and difficulty in abstract thinking. While olanzapine showed a symptom-specific effect on poor rapport at week 8, this effect demonstrated low stability. Overall, no robust symptom-specific medication effects emerged.

Although this is the first item-level examination of baseline network differences, our findings align with previous research showing no baseline network differences between remitters and non-remitters using composite PANSS domains, depressive symptoms, and cognitive schemas.^9^ Importantly, while these cross-sectional networks could not distinguish outcome groups, our longitudinal CLPN analyses revealed marked differences in symptom dynamics during treatment. This suggests that temporal symptom interactions may provide more meaningful insights into treatment response mechanisms than static baseline relationships alone.

At the global level, remitters showed a dense, highly interconnected network in the initial phase (baseline→week 2), where baseline symptoms relatively strongly predicted symptom improvement. This structured predictability suggests symptom evolution in remitters follows a systematic trajectory responsive to pharmacological intervention. Conversely, non-remitters exhibited a fragmented network with fewer and weaker predictive connections, indicating their baseline profiles were less informative for anticipating subsequent change. This reduced predictability may reflect a more heterogeneous or unstable illness course influenced by factors beyond direct antipsychotic effects.

These patterns diverged further during the subsequent treatment phase (week 2 → week 4). Among remitters, the symptom network became less densely connected, mirroring their clinical course of substantial early improvement followed by more gradual change. This aligns with prior research showing that the most pronounced therapeutic effects typically occur within the first two weeks of antipsychotic treatment.^42^ The observed reduction in network density may reflect a relative decoupling of symptom interactions as symptom severity declines, with individual symptoms becoming more independent.

In contrast, non-remitters showed a shift from an initially fragmented network to a more densely interconnected 1 in the week 2 → week 4 interval, reflecting a more complex and increasingly interdependent symptom landscape. Notably, all outgoing edges from positive symptoms in this network carried negative weights. Combined with the observed symptom trajectories—where positive symptoms generally continued to decline while negative and cognitive/disorganized symptoms increased—this pattern suggests an inverse relationship: reductions in positive symptoms at week 2 predicted increases in other symptom domains by week 4. This dynamic may reflect a trade-off during treatment, where improvement in positive symptoms is accompanied by a worsening of negative or cognitive symptoms. Such dynamics could point to emerging secondary negative symptoms, neurobiological compensation, or unintended effects of treatment itself.

Some specific findings warrant detailed discussion. During baseline→week 2 in remitters, poor impulse control showed the highest out-prediction value, suggesting that baseline behavioral regulation may facilitate early antipsychotic response, consistent with evidence linking impulse control to treatment engagement and adherence.^43^ While previous research demonstrated that poor impulse control predicts worse functional outcomes in early-onset psychosis,^44^ our network analysis extends this by revealing the temporal influence of impulse control on subsequent symptom severity. Delusions’ high in-prediction value in remitters suggests that this symptom serves as a central marker of early treatment response, with suspiciousness as its primary predictor. This aligns with research showing suspiciousness often precedes delusional thinking as part of an evolving "delusional mood,"^45^ suggesting paranoid symptoms may influence delusion improvement rates during treatment. Poor rapport's highest out-prediction value at week 2 → week 4 suggests that after initial improvement (baseline→week 2), improvement in social functioning facilitates positive changes in other symptoms, consistent with research showing lack of social contact predicted more severe negative symptoms.^46^ In previous CLPN research, poor rapport was also found to predict changes in other symptoms.^11^

Notable findings for non-remitters included minimal predictability for delusions at baseline→week 2, suggesting changes in delusions were not systematically related to prior symptom states. Combined with high in-prediction for disorientation and high out-prediction for disturbance of volition, this pattern may indicate a symptom profile that is less responsive to conventional dopamine blockade. During week 2 → week 4, difficulty in abstract thinking emerged as the strongest predictor in the network, indicating that persistent cognitive/disorganized impairments may shape broader symptom dynamics and potentially impact recovery trajectories. This aligns with perspectives positioning cognitive dysfunction as the core feature of schizophrenia^47^^,^^48^ and is consistent with research showing that disorganized symptoms—including abstract thinking deficits—predict treatment resistance.^49^ Taken together, these findings raise the possibility that non-remitters comprise a subgroup in which cognitive dysfunction contributes to sustained symptom interconnectedness that standard antipsychotics do not fully address. These findings underscore the need to investigate whether adjunctive interventions targeting cognitive domains might benefit patients who show limited early response to antipsychotic medication.

Our finding of a few differences between continuing amisulpride and switching to olanzapine on a symptom level aligns with the primary findings from the OPTiMiSE trial,^14^ which demonstrated no significant difference in overall remission rates between the 2 treatments or in total PANSS score reductions. Our item-level analysis revealed only one difference: at week 8, olanzapine demonstrated a larger effect than amisulpride on poor rapport, suggesting a potential targeted benefit in this symptom domain despite overall comparable efficacy profiles. However, it is important to emphasize that the link between treatment and poor rapport lacked statistical robustness in our stability analysis, indicating this finding should be interpreted with caution rather than as definitive evidence of a treatment-specific effect.

Collectively, our findings challenge how treatment efficacy is currently evaluated in psychotic disorders. Clinical trials continue to rely predominantly on static severity measures such as total PANSS scores, treating psychosis as a problem of overall symptom burden rather than acknowledging its dynamic, multidimensional structure. Our temporal network analyses demonstrate that symptom interactions evolve distinctly across treatment trajectories, dynamics missed by conventional sum scores. This suggests that future trials could benefit from incorporating network-based endpoints that capture evolving symptom interdependencies during treatment. However, the present findings are hypothesis-generating: whether remission is reliably associated with reduced density requires replication. Moreover, our analyses reflect group-averaged patterns; individual patients may show heterogeneous network responses. Person-specific network approaches, for example, using ecological momentary assessment, will be needed to inform personalized treatment targets.^50^ Together, these findings motivate future work to define, validate, and prespecify network-based markers of treatment response.

## Strengths and Limitations

A major strength of our study is its multi-layered network approach, which integrates cross-sectional and temporal analyses to better capture treatment response and resistance. CLPN enabled the analysis of non-stationary temporal symptom dynamics, a limitation of many traditional time-series models. Additionally, integrating NIA offered a novel way to assess differential medication effects at the symptom level, highlighting treatment targets often missed by conventional methods.

Limitations of this work include its hypothesis-generating rather than causality-confirming design. Network analyses identify temporal patterns between symptoms but cannot establish causal relationships without experimental manipulation. Moreover, CLPN models operate at the group level and do not disaggregate within-person dynamics from between-person differences, precluding individual-level inference. Data quality considerations include potential measurement noise in PANSS ratings. Although stability analyses were conducted, some of the greater heterogeneity observed in non-remitters may partially reflect measurement error rather than true symptom dynamics.^51^ Future work could further disentangle these effects by comparing observed networks to null models with predefined levels of measurement noise. In addition, no validated method currently exists to statistically compare CLPN networks, limiting formal inference. The relatively small phase II sample size may also have limited power to detect differential medication effects with NIA and likely contributed to the low stability of the olanzapine–poor rapport association. Finally, the scope of the present study was restricted to FEP and active antipsychotic treatment. Future research is therefore needed to determine whether similar temporal network dynamics characterize later illness stages, where longer illness duration and sustained treatment exposure may give rise to more stable or entrenched symptom interdependencies. Likewise, studies incorporating placebo or non-pharmacological control conditions would help clarify the relative contribution of specific medication effects versus non-specific processes such as expectancy or natural symptom fluctuation. Comparing network-level changes between pharmacological and psychosocial interventions could help determine whether distinct patterns of symptom reorganization emerge or replicate across treatment modalities.

## Conclusions

In conclusion, temporal symptom-network analysis reveals dynamic patterns in early FEP treatment that are not evident from cross-sectional baseline assessments. The distinct temporal network trajectories in remitters versus non-remitters suggest that monitoring of dynamic symptom interactions could help identify patients who may benefit from alternative or adjunctive strategies. The absence of robust medication-specific effects between amisulpride and olanzapine suggests these treatments may have more general therapeutic effects, though this finding is exploratory given the small sample size.

## Supplementary Material

Supplementary_Material_sbag016

## References

[1] Ventriglio A, Gentile A, Bonfitto I, et al. Suicide in the early stage of schizophrenia. *Front Psychiatry*. 2016;7:116. 27445872 10.3389/fpsyt.2016.00116 PMC4921745

[2] Jordan G, Lutgens D, Joober R, Lepage M, Iyer SN, Malla A. The relative contribution of cognition and symptomatic remission to functional outcome following treatment of a first episode of psychosis. *J Clin Psychiatry*. 2014;75:e566-e572. 10.4088/JCP.13m08606 25004197

[3] Borsboom D . A network theory of mental disorders. *World Psychiatry*. 2017;16:5-13. 10.1002/wps.20375 28127906 PMC5269502

[4] Blanchard MA, Heeren A. Ongoing and future challenges of the network approach to psychopathology: from theoretical conjectures to clinical translations. *Compr Clin Psychol*. 2022;11:32-46. 10.1016/B978-0-12-818697-8.00044-3

[5] van Borkulo C, Boschloo L, Borsboom D, Penninx BW, Waldorp LJ, Schoevers RA. Association of symptom network structure with the course of depression. *JAMA Psychiatry*. 2015;72:1219-1226. 10.1001/jamapsychiatry.2015.2079 26561400

[6] Zhou J, Liu S, Mayes TL, et al. The network analysis of depressive symptoms before and after two weeks of antidepressant treatment. *J Affect Disord*. 2022;299:126-134. 10.1016/j.jad.2021.11.059 34838606

[7] McElroy E, Napoleone E, Wolpert M, Patalay P. Structure and connectivity of depressive symptom networks corresponding to early treatment response. *EClinicalMedicine*. 2019;8:29-36. 10.1016/j.eclinm.2019.02.009 31193604 PMC6537518

[8] Esfahlani FZ, Sayama H, Visser KF, Strauss GP. Sensitivity of the positive and negative syndrome scale (PANSS) in detecting treatment effects via network analysis. *Innov Clin Neurosci*. 2017;14:59-67. 29410938 PMC5788252

[9] Piao YH, Yun JY, Nguyen TB, et al. Longitudinal symptom network structure in first-episode psychosis: a possible marker for remission. *Psychol Med*. 2022;52:3193-3201. 10.1017/S0033291720005280 33588966

[10] Sun Y, Zhang Y, Lu Z, et al. Longitudinal network analysis reveals interactive change of schizophrenia symptoms during acute antipsychotic treatment. *Schizophr Bull*. 2023;49:208-217. 10.1093/schbul/sbac131 36179110 PMC9810008

[11] Zhang Z, Huang B, Wu W, et al. Dynamics of symptom network in patients with first-episode schizophrenia: insight from the CNFEST project. *Asian J Psychiatry*. 2024;101:104202. 10.1016/j.ajp.2024.104202 39244845

[12] Blanken TF, Van Der Zweerde T, Van Straten A, Van Someren EJ, Borsboom D, Lancee J. Introducing network intervention analysis to investigate sequential, symptom-specific treatment effects: a demonstration in co-occurring insomnia and depression. *Psychother Psychosom*. 2019;88:52-54. 10.1159/000495045 30625483 PMC6469840

[13] Leucht S, Winter-van Rossum I, Heres S, et al. The optimization of treatment and management of schizophrenia in Europe (OPTiMiSE) trial: rationale for its methodology and a review of the effectiveness of switching antipsychotics. *Schizophr Bull*. 2015;41:549-558. 10.1093/schbul/sbv019 25786408 PMC4393704

[14] Kahn RS, van Rossum IW, Leucht S, et al. Amisulpride and olanzapine followed by open-label treatment with clozapine in first-episode schizophrenia and schizophreniform disorder (OPTiMiSE): a three-phase switching study. *Lancet Psychiatry*. 2018;5:797-807. 10.1016/S2215-0366(18)30252-9 30115598

[15] Sheehan DV, Lecrubier Y, Sheehan KH, et al. The MINI-international neuropsychiatric interview (MINI): the development and validation of a structured diagnostic psychiatric interview for DSM-IV and ICD-10. *J Clin Psychiatry*. 1998;59:22-33. 9881538

[16] Kay SR, Fiszbein A, Opler LA. The positive and negative syndrome scale (PANSS) for schizophrenia. *Schizophr Bull*. 1987;13:261-276. 3616518 10.1093/schbul/13.2.261

[17] Andreasen NC, Carpenter WT Jr, Kane JM, et al. Remission in schizophrenia: proposed criteria and rationale for consensus. *Am J Psychiatry*. 2005;162:441-449. 15741458 10.1176/appi.ajp.162.3.441

[18] Dal Santo F, García-Portilla MP, Fernández-Egea E, et al. The dimensional structure of the positive and negative syndrome scale in first-episode schizophrenia spectrum disorders: an exploratory graph analysis from the OPTiMiSE trial. *Schizophrenia.* 2024;10:81. 10.1038/s41537-024-00499-5 39349504 PMC11442741

[19] Christensen AP, Golino H. Estimating the stability of psychological dimensions via bootstrap exploratory graph analysis: a Monte Carlo simulation and tutorial. *Psych*. 2021;3:479-500.

[20] Friedman J, Hastie T, Tibshirani R. Sparse inverse covariance estimation with the graphical lasso. *Biostatistics.* 2008;9:432-441. 18079126 10.1093/biostatistics/kxm045 PMC3019769

[21] Epskamp S, Cramer AO, Waldorp LJ, Schmittmann VD, Borsboom D. Qgraph: network visualizations of relationships in psychometric data. *J Stat Softw*. 2012;48:1-18.

[22] Ho D, Imai K, King G, Stuart EA. MatchIt: nonparametric preprocessing for parametric causal inference. *J Stat Softw*. 2011;42:1-28. 10.18637/jss.v042.i08

[23] Dal Santo F, Fonseca-Pedrero E, García-Portilla MP, et al. Searching for bridges between psychopathology and real-world functioning in first-episode psychosis: a network analysis from the OPTiMiSE trial. *Eur Psychiatry*. 2022;65:e33. 10.1192/j.eurpsy.2022.25 35686446 PMC9251819

[24] Robinaugh DJ, Millner AJ, McNally RJ. Identifying highly influential nodes in the complicated grief network. *J Abnorm Psychol*. 2016;125:747-757. 10.1037/abn0000181 27505622 PMC5060093

[25] Epskamp S, Borsboom D, Fried EI. Estimating psychological networks and their accuracy: a tutorial paper. *Behav Res Methods*. 2018;50:195-212. 10.3758/s13428-017-0862-1 28342071 PMC5809547

[26] van Borkulo CD, van Bork R, Boschloo L, et al. Comparing network structures on three aspects: a permutation test. *Psychol Methods*. 2023;28:1273-1285. 10.1037/met0000476 35404628

[27] R Core Team . R: A Language and Environment for Statistical Computing. R Foundation for Statistical Computing, 2023. https://www.R-project.org/.

[28] Van Borkulo CD, Epskamp S, Jones P, Haslbeck J, Millner A. NetworkComparisonTest: statistical comparison of two networks based on three invariance measures (2.2.1) [computer software]. Published 2019. Available from: https://CRAN.R-project.org/package=NetworkComparisonTest

[29] Wysocki A, McCarthy I, van Bork R, Cramer AO . Cross-lagged panel networks. Adv Psychology. 2025;2:e739621.

[30] Kuismin M, Sillanpää MJ. Use of Wishart prior and simple extensions for sparse precision matrix estimation. *PLoS One*. 2016;11:e0148171. 10.1371/journal.pone.0148171 26828427 PMC4734711

[31] Savalei V, Brace JC, Fouladi RT. We need to change how we compute RMSEA for nested model comparisons in structural equation modeling. *Psychol Methods*. 2023;29:480-493. 10.1037/met0000537 36622720

[32] Rubin M, Bicki A, Papini S, Smits JAJ, Telch MJ, Gray JS. Distinct trajectories of depression symptoms in early and middle adolescence: preliminary evidence from longitudinal network analysis. *J Psychiatr Res*. 2021;142:198-203. 10.1016/j.jpsychires.2021.07.053 34365068

[33] Wang S, Chong ZY, Zhang C, Xu W. Longitudinal associations between anxiety and depressive symptoms in adolescence, early adulthood, and old age: cross-lagged panel network analyses. *Depress Anxiety*. 2024;2024:6205475. 40226714 10.1155/da/6205475 PMC11919059

[34] Zhao H, Zhou A. Longitudinal relations between non-suicidal self-injury and both depression and anxiety among senior high school adolescents: a cross-lagged panel network analysis. *PeerJ*. 2024;12:e18134. 10.7717/peerj.18134 39391828 PMC11466236

[35] Zhao Y, Liang K, Qu D, He Y, Ren Y, Chi X. Unraveling depressive symptom networks: a three-year longitudinal study among Chinese junior high school adolescents. *J Res Adolesc*. 2025;35:e13040. 10.1111/jora.13040 39582479

[36] Jaccard P . Étude comparative de la distribution florale dans Une portion des Alpes et des Jura. *Bull Soc Vaudoise Sci Nat*. 1901;37:547-579.

[37] Costa LDF . Further generalizations of the Jaccard Index. arXiv preprint arXiv:2110.09619. Published 2021

[38] Friedman J, Hastie T, Tibshirani R. Regularization paths for generalized linear models via coordinate descent. *J Stat Softw*. 2010;33:1-22. 20808728 PMC2929880

[39] Rosseel Y . Lavaan: an R package for structural equation modeling. *J Stat Softw*. 2012;48:1-36.

[40] Haslbeck JMB, Waldorp LJ . mgm: Estimating time-varying mixed graphical models in high-dimensional data. J Stat Software. 2020;93:1-46. 10.18637/jss.v093.i08

[41] Haslbeck JM, Waldorp LJ. How well do network models predict observations? On the importance of predictability in network models. *Behav Res Methods*. 2018;50:853-861. 10.3758/s13428-017-0910-x 28718088 PMC5880858

[42] Agid O, Seeman P, Kapur S. The "delayed onset" of antipsychotic action—an idea whose time has come and gone: 2004 innovations in neuropsychopharmacology award paper. *J Psychiatry Neurosci*. 2006;31:93-100. 16575424 PMC1413955

[43] Yang J, Ko YH, Paik JW, et al. Symptom severity and attitudes toward medication: impacts on adherence in outpatients with schizophrenia. *Schizophr Res*. 2012;134:226-231. 10.1016/j.schres.2011.11.008 22133906

[44] Remberk B, Bażyńska AK, Bronowska Z, et al. Which aspects of long-term outcome are predicted by positive and negative symptoms in early-onset psychosis? An exploratory eight-year follow-up study. *Psychopathology.* 2015;48:47-55. 10.1159/000366489 25471137

[45] Hermans K, van der Steen Y, Kasanova Z, et al. Temporal dynamics of suspiciousness and hallucinations in clinical high risk and first episode psychosis. *Psychiatry Res*. 2020;290:113039. 32460186 10.1016/j.psychres.2020.113039

[46] Millier A, Siegrist K, Amri I, Toumi M, Aballéa S. Social contacts reduce negative symptoms, especially emotional withdrawal in patients with schizophrenia. *Value Health*. 2014;17:A455. 10.1016/j.jval.2014.08.1244 27201263

[47] Kahn RS . On the origins of schizophrenia. *Am J Psychiatry*. 2020;177:291-297. 10.1176/appi.ajp.2020.20020147 32233682

[48] Kahn RS, Sommer IE, Murray RM, et al. Schizophrenia. *Schizophrenia Nat Rev Dis Primers*. 2015;1:15067. 27189524 10.1038/nrdp.2015.67

[49] Wold KF, Kreis IV, Åsbø G, et al. Long-term clinical recovery and treatment resistance in first-episode psychosis: a 10-year follow-up study. *Schizophrenia.* 2024;10:69. 39174576 10.1038/s41537-024-00489-7 PMC11341913

[50] Mansueto AC, Wiers RW, van Weert J, Schouten BC, Epskamp S. Investigating the feasibility of idiographic network models. *Psychol Methods*. 2023;28:1052-1066. 34990189 10.1037/met0000466

[51] de Ron J, Robinaugh DJ, Fried EI, et al. Quantifying and addressing the impact of measurement error in network models. *Behav Res Ther*. 2022;157:104163. 36030733 10.1016/j.brat.2022.104163 PMC10786122

