# Supplementary material for: Symptom Network Dynamics during Antipsychotic Treatment in First-Episode Psychosis
Source: Schizophr Bull. 2026 Mar 21;52(2):sbag016. doi: 10.1093/schbul/sbag016 (PMC13005116; doi:10.1093/schbul/sbag016)
Supplement: Supplementary_Material_sbag016 [file supplementary_material_sbag016.docx]

**Symptom Network Dynamics during Antipsychotic Treatment in First-Episode Psychosis**

**Supplementary materials**

--------------------------------------------------------------------------------------------------------

1. Comparing Temporal Networks
2. Complementary CLPN analysis in full sample
3. Supplementary Tables
4. Supplementary Figures
5. References

--------------------------------------------------------------------------------------------------------

1. **Comparing Temporal Networks**

The Network Comparison Test (NCT) is commonly used to compare symptom networks across groups in cross-sectional data. However, the NCT has not been validated for longitudinal networks, and its application to temporal network models remains methodologically uncertain. To address this limitation, and in line with previous Cross-Lagged Panel Network (CLPN) studies,^1-4^ we used two complementary approaches to compare remitter and non-remitter networks: (1) the Jaccard Index,^5,6^ and (2) the correlation between non-zero edge weights.

First, we calculated the Jaccard Index to measure structural similarity between networks. We created binary adjacency matrices where connections were coded as present (1) or absent (0). The Jaccard Index was calculated as: J = |A∩B| / |A∪B|, where A∩B represents the number of connections present in both networks, and A∪B represents the total number of unique connections across both networks. This yielded a value between 0 (no overlap) and 1 (complete overlap).

Second, to examine similarity in edge weight strength, we calculated the matrix correlation of non-zero edge weights. Specifically, we vectorized both adjacency matrices, containing the regression weights from the CLPN models, and calculated a Pearson correlation coefficient (r) between them. Importantly, we limited this calculation to non-zero edges present in at least one of the networks. This choice was made because including the large number of absent edges (zeros) would artificially inflate correlations, given the high sparsity typical of CLPN models. Focusing on non-zero edges provide a more accurate estimate of the similarity in connections that actually emerged during model estimation.

1. **Complementary CLPN analysis in full sample**

**Symptom severity over time**

Item-level symptom severity over time for the full sample shows an overall declining pattern, with P1 and P6 showing the steepest decline from baseline. Most items show a stronger decrease from baseline to week 2 (T0→T1) compared to week 2 to week 4 (T1→T2), where the decline becomes more gradual. Initial symptom severity varied considerably between items, with P1 starting highest (4.08) and items like G8 starting lowest (1.57) (Figure S9, standard deviations in Table S4).

**Model selection: testing cross-time constraints**

As a first step, we examined whether symptom dynamics remained consistent across time intervals during antipsychotic treatment by testing the imposition of cross-time constraints. For the full sample, the unconstrained model fitted the data well; 𝜒2 (𝑑𝑓 = 791) = 1013.3, *P* < .001; 𝑅𝑀𝑆𝐸𝐴 = 0.025 (0.020, 0.030); 𝐶𝐹𝐼 = 0.99, 𝑇𝐿𝐼 = 0.97 (robust statistics reported). The model with cross-time constraints imposed also showed a good fit; 𝜒2 (𝑑𝑓 = 1057) = 1482.2, *P* < .001; 𝑅𝑀𝑆𝐸𝐴 = 0.030 (0.026, 0.034); 𝐶𝐹𝐼 = 0.97, 𝑇𝐿𝐼 = 0.95.  The chi-square difference test found a significant difference, 𝜒𝑑𝑖𝑓𝑓2 (𝑑𝑓 = 266) = 468.9, *P* < .001, meaning that adding cross-time constraints significantly reduced model fit. Therefore, we used the unconstrained model for all subsequent analyses to accurately capture the temporal variations in symptom dynamics during antipsychotic treatment.

**Cross-Lagged Panel Network**

Figure S10 and S11, presents the directed symptom networks for the full sample across both time intervals (T0→T1 and T1→T2), along with the in-and-out prediction values. The directed edges represent temporal associations between symptoms after controlling for all other symptoms at baseline (for T0→T1) and week 2 (for T1→T2). To enhance interpretability, autoregressive edges were excluded from the plots (see Figure S12 for complete networks including autoregressive edges). The mean weight of the autoregressive edges was 0.48 for the T0→T1 network and 0.51 for the T1→T2 network (Figure S13).

At T0→T1, disorientation (G10) had the highest in-prediction value (R² = 0.20), being primarily influenced by unusual thought content (G9, β = -0.17), social withdrawal (N4, β = 0.16), suspiciousness/persecution (P6, β = 0.13), disturbance of volition (G13, β = -0.13), and difficulty in abstract thinking (N5, β = 0.10). Social withdrawal (N4) showed the highest out-prediction value (R² = 0.02), influencing hallucinatory behavior (P3, β = 0.08), stereotyped thinking (N7, β = 0.09), suspiciousness/persecution (P6, β = 0.11), poor impulse control (G14, β = 0.13), unusual thought content (G9, β = 0.14), disorientation (G10, β = 0.16), uncooperativeness (G8, β = 0.17), emotional withdrawal (N2, β = 0.19), and hostility (P7, β = 0.23).

At T1→T2, uncooperativeness (G8) had the highest in-prediction value (R² = 0.15), influenced by social withdrawal (N4, β = 0.10), preoccupation (G15, β = -0.13), hostility (P7, β = 0.15), and stereotyped thinking (N7, β = 0.17). Difficulty in abstract thinking (N5) had the highest out-prediction value (R² = 0.01), influencing suspiciousness/persecution (P6, β = 0.10), lack of spontaneity and flow in conversation (N6, β = 0.11), and disorientation (G10, β = 0.16).

Complete edge weight matrices for all networks are provided in Supplementary Tables S12 and S13, detailing all directed associations between PANSS items for each time interval. Bootstrapped confidence intervals around edge weights were moderate (see Figure S14).

1. **Supplementary Tables**

**Table S1.** Communities and Items of Redefined PANSS

| **Community** | **Items** |
| --- | --- |
| Positive | P1. Delusions |
|  | P3. Hallucinatory behavior |
|  | P6. Suspiciousness/persecution |
|  | G9. Unusual thought content |
| Cognitive/disorganized | P2. Conceptual disorganization |
|  | N5. Difficulty in abstract thinking |
|  | N7. Stereotyped thinking |
|  | G5. Mannerisms and posturing |
|  | G10. Disorientation |
|  | G11. Poor attention |
|  | G13. Disturbance of volition |
|  | G15. Preoccupation |
| Excited/aggressive | P7. Hostility |
|  | G8. Uncooperativeness |
|  | G14. Poor impulse control |
| Negative | N1. Blunted affect |
|  | N2. Emotional withdrawal |
|  | N3. Poor rapport |
|  | N4. Social withdrawal |
|  | N5. Difficulty in abstract thinking |
|  | N6. Lack of spontaneity and flow in conversation |
|  | G7. Motor retardation |

This table presents the 21 items retained from the original PANSS based on a previous network stability analysis study,^7^ organized by their respective symptom domains. Abbreviations: PANSS, Positive and Negative Syndrome Scale.

**Table S2.** Sample Characteristics for Phase I: Week 2 and 4

|  |  | **W2** |  |  | **W4** |  |
| --- | --- | --- | --- | --- | --- | --- |
|  | **Whole group**  **(N =386)** | **Remitters**  **(N = 243)** | **Non-**  **remitters**  **(N = 143)** | **Whole group**  **(N = 371)** | **Remitters**  **N = 250)** | **Non-**  **remitters**  **(N = 121)** |
| Age, M (SD) | 25.8 (6.1) | 26.4 (6.4) | 24.7 (5.4) | 25.7 (6.1) | 26.3 (6.3) | 24.5 (5.3) |
| Sex, male, N (%) | 278 (72.0%) | 170 (70.0%) | 108(75.5%) | 266 (71.7%) | 174 (69.6%) | 92 (76.0%) |
| Race, N (%)     White     Black     Asian     Other | 337(87.3%)  24 (6.2%)  14 (3.6%)  11 (2.8%) | 209 (86.0%)  17 (7.0%)  9 (3.7%)  8 (3.3%) | 128(89.5%)  7 (4.9%)  5 (3.5%)  3 (2.1%) | 325(87.6%)  21 (5.7%)  15 (4.0%)  10 (2.7%) | 215 (86.0%)  17 (6.8%)  10 (4.0%)  8 (3.2%) | 110 (90.9%)  4 (3.3%)  5 (4.1%)  2 (1.7%) |
| Disease type, N (%)    Schizophrenia    Schizoaffective    Schizophreniform | 199 (51.5%)  23 (6.0%)  164(42.5%) | 111(45.7%)  19 (7.8%)  113(46.5%) | 88 (61.5%)  4 (2.8%)  51 (35.7%) | 198(53.4%)  21 (5.7%)  152 (45%) | 117(46.8%)  19 (7.6%)  114(45.6%) | 81 (67%)  2 (1.7%)  38(31.4%) |
| Duration of current episode, months, M (SD) | 6.5 (6.4) | 6.1 (6.0) | 7.2 (6.9) | 6.6 (6.4),  N = 357 | 6.0 (6.0),  N = 239 | 7.7 (7.0),  N = 118 |
| Education, years, M (SD) | 12.3 (3.0) | 12.5 (3.1) | 12.0 (2.8) | 12.4 (3.0),  N = 365 | 12.5 (3.1),  N = 246 | 12.1 (2.7),  N =118 |
| PANSS total score^a^, M (SD) | 44.8 (14.6) | 39.3 (11.9) | 54.2 (14.4) | 40.9 (14.3) | 34.5 (9.2) | 53.9 (13.7) |

Abbreviations: PANSS, Positive and Negative Syndrome Scale.

ᵃTotal score of the 21-item PANSS version used in this study, based on a previous network stability analysis study.^7^

**Table S3.** Sample Characteristics for Phase II: Week 8 and 10

|  |  | **W8** |  |  | **W10** |  |
| --- | --- | --- | --- | --- | --- | --- |
|  | **Full sample**  **(N = 72)** | **Amisulpride**  **(N = 35)** | **Olanzapine**  **(N = 37)** | **Full sample**  **(N = 72)** | **Amisulpride**  **(N = 32)** | **Olanzapine**  **(N = 40)** |
| Age | 24.7 (5.6) | 25.2 (5.8) | 24.2 (5.5) | 24.7 (5.6) | 25.0 (5.8) | 24.4 (5.5) |
| Sex | 55 (76.4%) | 28 (80.0%) | 27 (73.0%) | 53 (73.6%) | 24 (75.0%) | 29 (72.5%) |
| Race, N (%)     White     Black     Asian     Other | 68 (94.4%)  1 (1.4%)  1 (1.4%)  2 (2.8%) | 33 (94.3%)  1 (2.9%)  0  1 (2.9%) | 35 (94.6%)  0  1 (2.7%)  1 (2.7%) | 68 (94.4%)  1 (1.4%)  2 (2.8%)  1 (1.4%) | 31 (96.9%)  0  1 (3.1%)  0 | 37 (92.5%)  1 (2.5%)  1 (2.5%)  1 (2.5%) |
| Disease type, N (%)     Schizophrenia     Schizoaffective     Schizophreniform | 49 (68.1%)  1 (1.4%)  22 (30.6%) | 27 (77.1)  0  8 (22.9%) | 22 (59.4%)  1 (2.7%)  14 (37.8%) | 48 (66.7%)  1 (1.4%)  23 (31.9%) | 23 (71.9%)  0  9 (28.1%) | 25 (62.5%)  1 (2.5%)  14 (35.0%) |
| Duration of current episode, months, M (SD) | 11.9 (2.6),  N = 69 | 12.6 (2.7),  N = 34 | 11.3 (2.4), N = 35 | 11.9 (2.5),  N = 70 | 12.7 (2.4) | 11.2 (2.3)  N = 38 |
| Education, years, M (SD) | 8.3 (7.3),  N = 70 | 9.9 (7.9),  N = 34 | 6.7 (6.4),  N = 36 | 7.8 (6.9),  N = 71 | 9.1 (7.6) | 6.7 (6.2),  N = 39 |
| PANSS total score^a^, M (SD) | 50.1 (14.0) | 53.2 (13.1) | 47.1 (14.4) | 48.7 (13.9) | 50.00 (14.6) | 47.7 (13.5) |

Abbreviations: PANSS, Positive and Negative Syndrome Scale.

ᵃTotal score of the 21-item PANSS version used in this study, based on a previous network stability analysis study.^7^

|  |  | **Baseline** |  |  | **W2** |  |  | **W4** |  |
| --- | --- | --- | --- | --- | --- | --- | --- | --- | --- |
| **Item** | **Full Sample**  **(N= 446)**  **M (SD)** | **Remitters**  **(N =250)**  **M (SD)** | **Non-**  **Remitters**  **(N = 196)**  **M (SD)** | **Full Sample**  **(N =386)**  **M (SD)** | **Remitters**  **(N = 243)**  **M (SD)** | **Non-**  **Remitters**  **(N =143)**  **M (SD)** | **Full Sample**  **(N =371)**  **M (SD)** | **Remitters**  **(N = 249)**  **M (SD)** | **Non-**  **Remitters**  **(N = 122)**  **M (SD)** |
| P1 | 4.08 (1.37) | 3.94 (1.39) | 4.26 (1.33) | 2.91 (1.38) | 2.54 (1.22) | 3.54 (1.41) | 2.43 (1.29) | 1.89 (0.88) | 3.55 (1.30) |
| P2 | 2.99 (1.43) | 2.73 (1.38) | 3.32 (1.42) | 2.32 (1.29) | 1.91 (1.04) | 3.01 (1.37) | 1.96 (1.12) | 1.55 (0.77) | 2.80 (1.25) |
| P3 | 3.20 (1.64) | 2.96 (1.60) | 3.49 (1.63) | 2.05 (1.38) | 1.69 (1.03) | 2.66 (1.67) | 1.75 (1.19) | 1.37 (0.73) | 2.52 (1.52) |
| P6 | 3.80 (1.42) | 3.77 (1.45) | 3.84 (1.38) | 2.75 (1.36) | 2.46 (1.22) | 3.23 (1.45) | 2.34 (1.24) | 1.92 (0.96) | 3.20 (1.48) |
| P7 | 1.83 (1.12) | 1.76 (1.20) | 1.92 (1.20) | 1.57 (0.97) | 1.42 (0.78) | 1.81 (1.20) | 1.41 (0.88) | 1.20 (0.53) | 1.84 (1.23) |
| N1 | 2.89  (1.46) | 2.70  (1.42) | 3.13  (1.47) | 2.57  (1.36) | 2.22  (1.17) | 3.17  (1.45) | 2.44  (1.21) | 2.04  (0.89) | 3.26  (1.36) |
| N2 | 2.88  (1.39) | 2.61  (1.31) | 3.23  (1.41) | 2.59  (1.36) | 2.23  (1.22) | 3.20  (1.36) | 2.43  (1.24) | 2.06  (1.00) | 3.21  (1.32) |
| N3 | 2.56  (1.37) | 2.38  (1.29) | 2.79  (1.43) | 2.21  (1.25) | 1.93  (1.07) | 2.69  (1.39) | 2.09  (1.18) | 1.78  (0.92) | 2.74  (1.37) |
| N4 | 3.01  (1.52) | 2.72  (1.44) | 3.37  (1.55) | 2.65  (1.42) | 2.28  (1.23) | 3.29  (1.48) | 2.45  (1.30) | 2.00  (0.88) | 3.36  (1.53) |
| N5 | 3.04 (1.48) | 2.84 (1.42) | 3.30 (1.52) | 2.68 (1.42) | 2.35 (1.26) | 3.24 (1.50) | 2.46 (1.36) | 2.14 (1.21) | 3.11 (1.43) |
| N6 | 2.51 (1.46) | 2.38 (1.35) | 2.68 (1.57) | 2.32 (1.31) | 2.04 (1.10) | 2.79 (1.50) | 2.27 (1.25) | 1.89 (0.89) | 3.05 (1.50) |
| N7 | 2.49 (1.29) | 2.26 (1.20) | 2.77 (1.35) | 2.08 (1.19) | 1.71 (0.98) | 2.70 (1.26) | 1.87 (1.10) | 1.57 (0.83) | 2.49 (1.31) |
| G5 | 1.69 (1.08) | 1.54 (0.97) | 1.89 (1.19) | 1.56 (0.96) | 1.35 (0.74) | 1.92 (1.15) | 1.46 (0.84) | 1.24 (0.55) | 1.92 (1.10) |
| G7 | 1.96 (1.17) | 1.90 (1.14) | 2.03 (1.22) | 1.85 (1.05) | 1.63 (0.87) | 2.24 (1.21) | 1.80 (1.01) | 1.56 (0.83) | 2.29 (1.18) |
| G8 | 1.57 (0.99) | 1.45 (0.85) | 1.71 (1.12) | 1.39 (0.80) | 1.24 (0.61) | 1.64 (1.00) | 1.29 (0.77) | 1.14 (0.50) | 1.60 (1.08) |
| G9 | 3.20 (1.33) | 3.06 (1.35) | 3.38 (1.30) | 2.32 (1.17) | 2.07 (1.05) | 2.74 (1.24) | 2.09 (1.12) | 1.71 (0.84) | 2.86 (1.23) |
| G10 | 1.70 (1.02) | 1.56 (0.91) | 1.87 (1.12) | 1.41 (0.79) | 1.30 (0.67) | 1.61 (0.92) | 1.34 (0.70) | 1.24 (0.58) | 1.55 (0.85) |

**Table S4.** PANSS Item Scores at Each Time Point for the Full Sample, Remitters, and Non-Remitters

**Table S4** (continued)

| G11 | 2.58 (1.27) | 2.49 (1.26) | 2.69 (1.28) | 2.14 (1.16) | 1.94 (1.06) | 2.48 (1.26) | 1.90 (1.00) | 1.70 (0.84) | 2.30 (1.17) |
| --- | --- | --- | --- | --- | --- | --- | --- | --- | --- |
| G13 | 2.28 (1.27) | 2.16 (1.22) | 2.42 (1.32) | 1.90 (1.12) | 1.67 (0.97) | 2.29 (1.26) | 1.82 (1.03) | 1.55 (0.82) | 2.37 (1.19) |
| G14 | 1.93 (1.20) | 1.86 (1.17) | 2.02 (1.24) | 1.55 (0.96) | 1.46 (0.88) | 1.70 (1.06) | 1.44 (0.84) | 1.27 (0.61) | 1.80 (1.10) |
| G15 | 2.54 (1.39) | 2.40 (1.39) | 2.73 (1.37) | 2.13 (1.19) | 1.88 (1.01) | 2.57 (1.33) | 1.93 (1.12) | 1.67 (0.86) | 2.45 (1.38) |

Abbreviations: P1, delusions; P2, conceptual disorganization; P3, hallucinatory behavior; P6, suspiciousness/persecution; P7, hostility; N1, blunted affect; N2, emotional withdrawal; N3, poor rapport; N4, social withdrawal; N5, difficulty in abstract thinking; N6, lack of spontaneity and flow in conversation; N7, stereotyped thinking; G5, mannerisms and posturing; G7, motor retardation; G8, uncooperativeness; G9, unusual thought content; G10, disorientation; G11, poor attention; G13, disturbance of volition; G14, poor impulse control; G15, preoccupation.

**Table S5**. PANSS Item Scores at Each Time Point for the Full Sample, Amisulpride and Olanzapine Groups

|  |  | **W6** |  |  | **W8** |  |  | **W10** |  |
| --- | --- | --- | --- | --- | --- | --- | --- | --- | --- |
| **Item** | **Full sample**  **(N = 85)** | **Amisul**  **pride**  **(N = 43)** | **Olanza**  **pine**  **(N = 42)** | **Full sample**  **(N = 72)** | **Amisul**  **pride**  **(N = 35)** | **Olanza**  **pine**  **(N = 37)** | **Full sample**  **(N = 72)** | **Amisul**  **pride**  **(N = 32)** | **Olanza**  **pine**  **(N = 40)** |
| P1 | 3.42  (1.34) | 3.40  (1.22) | 3.45  (1.48) | 3.24  (1.31) | 3.09 (1.22) | 3.38  (1.38) | 2.99  (1.38) | 2.72  (1.44) | 3.20  (1.30) |
| P2 | 2.76  (1.28) | 2.77  (1.31) | 2.76  (1.27) | 2.49  (1.23) | 2.71 (1.18) | 2.27  (1.26) | 2.43  (1.36) | 2.38  (1.26) | 2.48  (1.45) |
| P3 | 2.40  (1.58) | 2.37  (1.56) | 2.43  (1.63) | 2.43  (1.56 | 2.26 (1.52) | 2.59 (1.61) | 2.21  (1.53) | 2.07  (1.44) | 2.33  (1.61) |
| P6 | 3.20  (1.40) | 3.00  (1.15) | 3.41  (1.61) | 2.90  (1.56) | 2.80  (1.26) | 3.00  (1.45) | 2.90  (1.47) | 2.66  (1.31) | 3.10  (1.58) |
| P7 | 1.73  (1.25) | 1.63  (1.15) | 1.83  (1.34) | 1.64  (1.04) | 1.77  (1.06) | 1.51  (1.02) | 1.68  (1.07) | 1.81  (1.06) | 1.58  (1.08) |
| N1 | 3.15  (1.31) | 3.26  (1.33) | 3.05  (1.31) | 2.97  (1.38) | 3.23  (1.33) | 2.73  (1.41) | 2.97  (1.35) | 3.13  (1.29) | 2.85  (1.41) |
| N2 | 3.08  (1.32) | 3.23  (1.27) | 2.93  (1.37) | 3.00  (1.36) | 3.31  (1.25) | 2.70  (1.41) | 3.07  (1.22) | 3.13  (1.18) | 3.03  (1.27) |
| N3 | 2.56  (1.35) | 2.58  (1.30) | 2.55  (1.42) | 2.46  (1.40) | 2.91  (1.34) | 2.02  (1.41) | 2.42  (1.37) | 2.69  (1.38) | 2.20  (1.34) |
| N4 | 3.29  (1.45) | 3.55  (1.48) | 3.02  (1.39) | 3.13  (1.50) | 3.40  (1.42) | 2.86  (1.55) | 3.11  (1.51) | 3.19  (1.62) | 3.05  (1.43) |
| N5 | 3.18  (1.36) | 3.30  (1.46) | 3.05  (1.27) | 3.14  (1.28) | 3.29  (1.38) | 3.00  (1.18) | 2.83  (1.26) | 2.84  (1.37) | 2.83  (1.17) |
| N6 | 2.89  (1.49) | 3.02  (1.49) | 2.76  (1.51) | 2.78  (1.42) | 3.17  (1.27) | 2.41  (1.46) | 2.65  (1.29) | 3.00  (1.22) | 2.38  (1.29) |
| N7 | 2.44  (1.26) | 2.44  (1.18) | 2.43  (1.35) | 2.21  (1.29) | 2.34  (1.19) | 2.08  (1.38) | 2.18  (1.27) | 2.13  (1.01) | 2.23  (1.46) |
| G5 | 1.87  (1.21) | 2.02  (1.32) | 1.71 (1.09) | 1.68  (1.00) | 1.83  (1.04) | 1.54  (0.96) | 1.63  (0.85) | 1.59  (0.80) | 1.65 (0.89) |
| G7 | 2.16  (1.14) | 2.37  (1.20) | 1.95  (1.06) | 2.18  (1.12) | 2.34  (1.06) | 2.03  (1.17) | 2.06  (1.11) | 2.13  (1.16) | 2.00  (1.09) |
| G8 | 1.52  (1.04) | 1.53  (1.10) | 1.50  (0.99) | 1.51  (0.93) | 1.60  (0.98) | 1.43  (0.90) | 1.53  (1.01) | 1.66  (0.90) | 1.43  (1.08) |
| G9 | 2.69  (1.25) | 2.88  (1.20) | 2.50  (1.29) | 2.46  (1.14) | 2.60  (1.17) | 2.32  (1.11) | 2.42  (1.20) | 2.47  (1.24) | 2.38  (1.17) |
| G10 | 1.56  (0.84) | 1.58  (0.85) | 1.55  (0.83) | 1.60  (0.91) | 1.63  (0.97) | 1.57  (0.87) | 1.58  (0.99) | 1.72  (1.14) | 1.48  (0.85) |

**Table S5** (continued)

| G11 | 2.15  (1.09) | 2.30  (1.15) | 2.00  (1.01) | 2.33  (1.15) | 2.43  (1.04) | 2.24  (1.26) | 2.25  (1.12) | 2.50  (1.08) | 2.05  (1.13) |  |  |
| --- | --- | --- | --- | --- | --- | --- | --- | --- | --- | --- | --- |
| G13 | 2.33  (1.25) | 2.47  (1.16) | 2.19  (1.33) | 1.99  (1.28) | 2.20  (1.23) | 1.78  (1.32) | 1.89  (1.19) | 2.09  (1.12) | 1.73  (1.40) |  |  |
| G14 | 1.69  (1.10) | 1.53  (1.05) | 1.86  (1.14) | 1.61  (1.08) | 1.72  (1.15) | 1.49  (1.02) | 1.64  (1.03) | 1.75  (1.05) | 1.55  (1.01) |  |  |
| G15 | 2.33  (1.35) | 2.42  (1.38) | 2.24  (1.32) | 2.36  (1.31) | 2.57  (1.24) | 2.16  (1.36) | 2.28  (1.27) | 2.34  (1.29) | 2.23  (1.27) |  |  |

Abbreviations: P1, delusions; P2, conceptual disorganization; P3, hallucinatory behavior; P6, suspiciousness/persecution; P7, hostility; N1, blunted affect; N2, emotional withdrawal; N3, poor rapport; N4, social withdrawal; N5, difficulty in abstract thinking; N6, lack of spontaneity and flow in conversation; N7, stereotyped thinking; G5, mannerisms and posturing; G7, motor retardation; G8, uncooperativeness; G9, unusual thought content; G10, disorientation; G11, poor attention; G13, disturbance of volition; G14, poor impulse control; G15, preoccupation.

**Table S6.** Complete Edge Weight Matrix for Symptom Relationships in Remitters at Baseline

|  | **P1** | **P2** | **P3** | **P6** | **P7** | **N1** | **N2** | **N3** | **N4** | **N5** | **N6** | **N7** | **G5** | **G7** | **G8** | **G9** | **G10** | **G11** | **G13** | **G14** | **G15** |
| --- | --- | --- | --- | --- | --- | --- | --- | --- | --- | --- | --- | --- | --- | --- | --- | --- | --- | --- | --- | --- | --- |
| P1 | - |  |  |  |  |  |  |  |  |  |  |  |  |  |  |  |  |  |  |  |  |
| P2 | .00 | - |  |  |  |  |  |  |  |  |  |  |  |  |  |  |  |  |  |  |  |
| P3 | .18 | .00 | - |  |  |  |  |  |  |  |  |  |  |  |  |  |  |  |  |  |  |
| P6 | .43 | .00 | .03 | - |  |  |  |  |  |  |  |  |  |  |  |  |  |  |  |  |  |
| P7 | .00 | .00 | .00 | .07 | - |  |  |  |  |  |  |  |  |  |  |  |  |  |  |  |  |
| N1 | .00 | .00 | .00 | .00 | .00 | - |  |  |  |  |  |  |  |  |  |  |  |  |  |  |  |
| N2 | .00 | .00 | .00 | .00 | .00 | .18 | - |  |  |  |  |  |  |  |  |  |  |  |  |  |  |
| N3 | -.04 | .08 | .00 | .00 | .00 | .21 | .09 | - |  |  |  |  |  |  |  |  |  |  |  |  |  |
| N4 | .00 | .00 | .03 | .03 | .00 | .14 | .54 | .01 | - |  |  |  |  |  |  |  |  |  |  |  |  |
| N5 | .00 | .02 | .00 | .00 | .00 | .00 | .00 | .00 | .00 | - |  |  |  |  |  |  |  |  |  |  |  |
| N6 | .00 | .03 | .00 | .00 | .00 | .14 | .02 | .37 | .00 | .15 | - |  |  |  |  |  |  |  |  |  |  |
| N7 | .03 | .12 | .00 | .00 | .00 | .00 | .00 | .15 | .00 | .15 | .00 | - |  |  |  |  |  |  |  |  |  |
| G5 | .00 | .10 | .00 | .00 | .00 | .00 | .03 | .00 | .00 | .06 | .03 | .02 | - |  |  |  |  |  |  |  |  |
| G7 | .00 | .00 | .00 | .00 | .00 | .27 | .00 | .00 | .04 | .00 | .06 | .00 | .13 | - |  |  |  |  |  |  |  |
| G8 | .00 | .00 | .00 | .02 | .21 | .00 | .00 | .13 | .00 | .00 | .06 | .00 | .00 | .00 | - |  |  |  |  |  |  |
| G9 | .36 | .10 | .08 | .13 | .00 | .00 | .00 | .00 | .01 | .04 | .00 | .05 | .00 | .00 | .02 | - |  |  |  |  |  |
| G10 | .00 | .01 | .00 | .00 | .00 | .00 | .00 | .00 | .00 | .15 | .00 | .00 | .12 | .00 | .00 | .00 | - |  |  |  |  |
| G11 | .00 | .29 | .04 | .02 | .00 | .00 | .02 | .00 | .00 | .00 | .01 | .01 | .00 | .00 | .00 | .00 | .03 | - |  |  |  |
| G13 | .00 | .09 | .05 | .00 | .00 | .04 | .15 | .00 | .00 | .00 | .14 | .09 | .08 | .07 | .09 | .01 | .00 | .21 | - |  |  |
| G14 | .11 | .00 | .00 | .00 | .35 | .00 | .00 | .00 | .00 | .00 | .00 | .00 | .00 | -.02 | .15 | .14 | .00 | .00 | .06 | - |  |
| G15 | .03 | .04 | .02 | .06 | .01 | .00 | .10 | .05 | .06 | .00 | .00 | .18 | .06 | .00 | .06 | .01 | .00 | .16 | .07 | .00 | - |

Abbreviations: P1, delusions; P2, conceptual disorganization; P3, hallucinatory behavior; P6, suspiciousness/persecution; P7, hostility; N1, blunted affect; N2, emotional withdrawal; N3, poor rapport; N4, social withdrawal; N5, difficulty in abstract thinking; N6, lack of spontaneity and flow in conversation; N7, stereotyped thinking; G5, mannerisms and posturing; G7, motor retardation; G8, uncooperativeness; G9, unusual thought content; G10, disorientation; G11, poor attention; G13, disturbance of volition; G14, poor impulse control; G15, preoccupation.

**Table S7.** Complete Edge Weight Matrix for Symptom Relationships in Non-Remitters at Baseline

|  | **P1** | **P2** | **P3** | **P6** | **P7** | **N1** | **N2** | **N3** | **N4** | **N5** | **N6** | **N7** | **G5** | **G7** | **G8** | **G9** | **G10** | **G11** | **G13** | **G14** | **G15** |
| --- | --- | --- | --- | --- | --- | --- | --- | --- | --- | --- | --- | --- | --- | --- | --- | --- | --- | --- | --- | --- | --- |
| P1 | - |  |  |  |  |  |  |  |  |  |  |  |  |  |  |  |  |  |  |  |  |
| P2 | .00 | - |  |  |  |  |  |  |  |  |  |  |  |  |  |  |  |  |  |  |  |
| P3 | .17 | .00 | - |  |  |  |  |  |  |  |  |  |  |  |  |  |  |  |  |  |  |
| P6 | .39 | .00 | .04 | - |  |  |  |  |  |  |  |  |  |  |  |  |  |  |  |  |  |
| P7 | .00 | .00 | .00 | .10 | - |  |  |  |  |  |  |  |  |  |  |  |  |  |  |  |  |
| N1 | .00 | .00 | .00 | -.01 | .00 | - |  |  |  |  |  |  |  |  |  |  |  |  |  |  |  |
| N2 | .00 | .05 | .00 | .00 | .00 | .22 | - |  |  |  |  |  |  |  |  |  |  |  |  |  |  |
| N3 | .00 | .05 | .00 | .00 | .00 | .21 | .11 | - |  |  |  |  |  |  |  |  |  |  |  |  |  |
| N4 | .00 | .00 | .00 | .00 | .10 | .00 | .48 | .04 | - |  |  |  |  |  |  |  |  |  |  |  |  |
| N5 | .00 | .05 | .03 | .00 | .00 | .00 | .00 | .00 | .00 | - |  |  |  |  |  |  |  |  |  |  |  |
| N6 | -.05 | .02 | .00 | .00 | .00 | .15 | .00 | .45 | .09 | .10 | - |  |  |  |  |  |  |  |  |  |  |
| N7 | .07 | .22 | .00 | .00 | .00 | .00 | .01 | .00 | .04 | .01 | .04 | - |  |  |  |  |  |  |  |  |  |
| G5 | .00 | .03 | .00 | .00 | .00 | .08 | .00 | .04 | .00 | .00 | .00 | .00 | - |  |  |  |  |  |  |  |  |
| G7 | .00 | .01 | .00 | -.03 | .00 | .28 | .05 | .00 | .02 | .00 | .00 | .00 | .09 | - |  |  |  |  |  |  |  |
| G8 | .00 | .04 | .00 | .00 | .26 | .00 | .00 | .10 | .00 | .00 | .07 | .00 | .07 | .00 | - |  |  |  |  |  |  |
| G9 | .34 | .00 | .08 | .00 | .00 | .00 | .00 | .00 | .00 | .00 | .00 | .02 | .00 | .00 | .00 | - |  |  |  |  |  |
| G10 | .00 | .00 | .00 | .00 | .00 | .00 | .00 | .00 | .00 | .18 | .00 | .00 | .00 | .00 | .00 | .00 | - |  |  |  |  |
| G11 | .00 | .19 | .00 | .00 | .04 | .00 | .01 | .00 | .00 | .00 | .02 | .03 | .02 | .06 | .05 | .00 | .06 | - |  |  |  |
| G13 | .00 | .12 | .04 | .00 | .03 | .00 | .04 | .01 | .00 | .00 | .03 | .00 | .11 | .00 | .00 | .00 | .00 | .26 | - |  |  |
| G14 | .08 | .00 | .13 | .00 | .32 | .00 | .00 | .00 | .00 | .08 | .00 | .02 | .00 | .00 | .01 | .00 | .05 | .00 | .03 | - |  |
| G15 | .01 | .03 | .03 | .00 | .00 | .06 | .11 | .02 | .00 | .05 | .00 | .15 | .12 | .00 | .00 | .12 | .00 | .16 | .14 | .00 | - |

Abbreviations: P1, delusions; P2, conceptual disorganization; P3, hallucinatory behavior; P6, suspiciousness/persecution; P7, hostility; N1, blunted affect; N2, emotional withdrawal; N3, poor rapport; N4, social withdrawal; N5, difficulty in abstract thinking; N6, lack of spontaneity and flow in conversation; N7, stereotyped thinking; G5, mannerisms and posturing; G7, motor retardation; G8, uncooperativeness; G9, unusual thought content; G10, disorientation; G11, poor attention; G13, disturbance of volition; G14, poor impulse control; G15, preoccupation.

**Table S8.** Complete Edge Weight Matrix for Symptom Interactions in Remitters from Baseline to Week 2 (T0→T1)

|  | **P1** | **P2** | **P3** | **P6** | **P7** | **N1** | **N2** | **N3** | **N4** | **N5** | **N6** | **N7** | **G5** | **G7** | **G8** | **G9** | **G10** | **G11** | **G13** | **G14** | **G15** |
| --- | --- | --- | --- | --- | --- | --- | --- | --- | --- | --- | --- | --- | --- | --- | --- | --- | --- | --- | --- | --- | --- |
| P1 | .39 | .00 | .00 | .00 | .00 | .00 | .00 | .00 | .00 | .00 | .00 | .00 | .00 | .00 | .00 | .00 | .00 | .00 | .00 | .00 | .00 |
| P2 | .00 | .47 | .00 | .00 | -.14 | .00 | -.16 | .00 | -.11 | .00 | .00 | .00 | .00 | .00 | .00 | .00 | .00 | .00 | .00 | -.13 | .00 |
| P3 | .00 | .00 | .51 | .00 | .00 | .00 | -.08 | .00 | .00 | .00 | -.12 | .00 | .00 | .00 | .00 | .00 | .00 | .00 | .00 | .00 | -.10 |
| P6 | .18 | .00 | .00 | .40 | .00 | .00 | .00 | .00 | .00 | .00 | .00 | .00 | .00 | .00 | .00 | .00 | .00 | .00 | .00 | .00 | .00 |
| P7 | .00 | .00 | .00 | .00 | .49 | .00 | .00 | .00 | .00 | .00 | .00 | .00 | .00 | .00 | .00 | .00 | .00 | .00 | .00 | .00 | .00 |
| N1 | .00 | .00 | .00 | .00 | .00 | .53 | .00 | .00 | .00 | .00 | .00 | .00 | .00 | .00 | .00 | .00 | .00 | .00 | .00 | .00 | .00 |
| N2 | .00 | .00 | .00 | .00 | .00 | .00 | .32 | .00 | .25 | .00 | .00 | .00 | .00 | .00 | .00 | .00 | .00 | .00 | .00 | .00 | .00 |
| N3 | -.15 | .00 | .00 | .00 | .00 | .00 | .00 | .61 | .00 | .00 | .17 | .00 | .00 | .00 | .00 | -.17 | .00 | .00 | .16 | .00 | .00 |
| N4 | .00 | .00 | .00 | .15 | .10 | .00 | .22 | .00 | .48 | .00 | .00 | .00 | .00 | .00 | .00 | .00 | .00 | .00 | .00 | .00 | .00 |
| N5 | .10 | .00 | .00 | .00 | .00 | .00 | .08 | .00 | .19 | .61 | .00 | .00 | .00 | .00 | .00 | .00 | .00 | .00 | .00 | .00 | .00 |
| N6 | .00 | .00 | .00 | .00 | .00 | .00 | .00 | .00 | .00 | .00 | .42 | .00 | .00 | .00 | .00 | .00 | .00 | .00 | .00 | .00 | .00 |
| N7 | .00 | .18 | .00 | .00 | .00 | .11 | .00 | .00 | .00 | .00 | .00 | .47 | .00 | .00 | .00 | .00 | .00 | .00 | .00 | .00 | .00 |
| G5 | .00 | .12 | .00 | .00 | .11 | .00 | .00 | .00 | .00 | .00 | .00 | .00 | .55 | .00 | .00 | .11 | .00 | .00 | .00 | .00 | .00 |
| G7 | .00 | -.11 | .00 | .00 | .00 | .00 | .00 | .00 | .00 | .00 | .00 | .00 | .00 | .47 | .00 | .00 | .00 | .00 | .00 | .00 | .00 |
| G8 | .00 | .00 | .00 | .00 | .00 | .00 | .00 | .00 | .00 | .00 | .00 | .00 | .00 | .00 | .47 | .00 | .00 | .10 | .00 | .00 | .00 |
| G9 | .00 | -.11 | .00 | .00 | .00 | .00 | .00 | .00 | .00 | .00 | .00 | .00 | .00 | .00 | .00 | .38 | -.12 | .00 | .00 | .00 | .00 |
| G10 | .00 | .00 | .00 | .13 | .00 | .00 | .00 | .00 | .00 | .14 | .00 | .00 | .12 | .00 | .00 | .00 | .48 | .00 | .00 | .23 | .00 |
| G11 | .00 | .00 | .00 | .00 | .00 | .00 | .00 | .00 | .00 | -.17 | .00 | .00 | .00 | .00 | .00 | .00 | .00 | .43 | .00 | .00 | .00 |
| G13 | .15 | .00 | .00 | .00 | .00 | .00 | .00 | .00 | .00 | .00 | .16 | .00 | .00 | .00 | .00 | .00 | .00 | .00 | .46 | .00 | .00 |
| G14 | .12 | .16 | .00 | .18 | .17 | .00 | .00 | .00 | .00 | .00 | .00 | .00 | .00 | .00 | .13 | .19 | .00 | .00 | .00 | .53 | .00 |
| G15 | .09 | .00 | .00 | .00 | .00 | .00 | .00 | .00 | .00 | .11 | .00 | .15 | .00 | .00 | .00 | .13 | .00 | .18 | .00 | .00 | .57 |

Abbreviations: P1, delusions; P2, conceptual disorganization; P3, hallucinatory behavior; P6, suspiciousness/persecution; P7, hostility; N1, blunted affect; N2, emotional withdrawal; N3, poor rapport; N4, social withdrawal; N5, difficulty in abstract thinking; N6, lack of spontaneity and flow in conversation; N7, stereotyped thinking; G5, mannerisms and posturing; G7, motor retardation; G8, uncooperativeness; G9, unusual thought content; G10, disorientation; G11, poor attention; G13, disturbance of volition; G14, poor impulse control; G15, preoccupation.

**Table S9.** Complete Edge Weight Matrix for Symptom Interactions in Remitters from Week 2 to Week 4 (T1→T2)

|  | **P1** | **P2** | **P3** | **P6** | **P7** | **N1** | **N2** | **N3** | **N4** | **N5** | **N6** | **N7** | **G5** | **G7** | **G8** | **G9** | **G10** | **G11** | **G13** | **G14** | **G15** |
| --- | --- | --- | --- | --- | --- | --- | --- | --- | --- | --- | --- | --- | --- | --- | --- | --- | --- | --- | --- | --- | --- |
| P1 | .49 | .00 | .00 | .00 | .00 | .00 | .00 | .00 | .00 | .00 | .00 | .00 | .00 | .00 | .00 | .00 | .00 | .00 | .00 | .00 | .00 |
| P2 | .00 | .63 | -.12 | .00 | .00 | .00 | .00 | .00 | .00 | .00 | .00 | .00 | .00 | .00 | .00 | .00 | .00 | .00 | .00 | .00 | .00 |
| P3 | .00 | .00 | .55 | .00 | .00 | .00 | .00 | .00 | .00 | .00 | .00 | .00 | .00 | .00 | .00 | .00 | .00 | .00 | .00 | .00 | .00 |
| P6 | .00 | .00 | .00 | .56 | .00 | -.08 | .00 | .00 | .00 | .00 | .00 | .00 | .00 | .00 | .00 | .00 | .00 | -.10 | .00 | .00 | .00 |
| P7 | .00 | .00 | .00 | .00 | .49 | .00 | .00 | .00 | .00 | .00 | -.09 | .00 | .00 | .00 | .00 | .00 | .00 | .00 | .00 | .00 | .00 |
| N1 | .00 | .00 | .00 | .00 | .00 | .50 | .00 | .00 | .00 | .00 | .00 | .00 | .00 | .00 | .00 | .00 | .00 | .00 | .00 | -.12 | .00 |
| N2 | .00 | .00 | -.16 | .00 | .00 | .21 | .47 | .00 | .19 | .00 | .00 | .00 | .00 | .00 | .00 | .00 | .00 | .00 | .00 | .00 | .00 |
| N3 | .00 | .00 | .16 | .00 | .00 | .00 | .00 | .42 | .00 | .00 | .00 | .13 | .00 | .00 | .22 | .00 | .00 | .00 | .00 | .00 | .00 |
| N4 | .00 | .00 | .00 | .00 | .00 | .00 | .13 | .00 | .38 | .11 | .00 | .00 | .00 | .00 | .00 | .00 | .00 | .00 | .00 | .00 | .00 |
| N5 | .00 | .00 | .00 | .11 | .00 | .00 | .00 | .00 | .00 | .61 | .13 | .10 | .00 | .00 | .00 | .00 | .00 | .00 | .00 | .00 | .00 |
| N6 | .00 | .00 | .00 | .00 | .00 | .00 | .00 | .00 | .00 | .00 | .49 | .00 | .00 | -.21 | .00 | .00 | .00 | .00 | .00 | .00 | .00 |
| N7 | .00 | .00 | .00 | .00 | .00 | .13 | .00 | .00 | .00 | .00 | .00 | .48 | .00 | .00 | .00 | .00 | .00 | .00 | .00 | .00 | .00 |
| G5 | .00 | .12 | .00 | .00 | .00 | .00 | .00 | .00 | .00 | .14 | .00 | .00 | .53 | .00 | .00 | .00 | .00 | .00 | .00 | .00 | .00 |
| G7 | .00 | .00 | .00 | .00 | .00 | .00 | .00 | .00 | .00 | .00 | .00 | .00 | .00 | .49 | .00 | .00 | .00 | .00 | .00 | .00 | .00 |
| G8 | .00 | .00 | .11 | .00 | .00 | .00 | .11 | .00 | .00 | .00 | .00 | .00 | .00 | .00 | .34 | .00 | .00 | .00 | .00 | .00 | .00 |
| G9 | .18 | .00 | .00 | .00 | .00 | .00 | .00 | .00 | .00 | .00 | .00 | .00 | .00 | .00 | .00 | .61 | .00 | .00 | .00 | .00 | .00 |
| G10 | .00 | .00 | .00 | .00 | .00 | .00 | .00 | .00 | .00 | .00 | .00 | .00 | .00 | .00 | .00 | .14 | .56 | .00 | .00 | .00 | .00 |
| G11 | .00 | .00 | .00 | .00 | .00 | .00 | .00 | .00 | .00 | .00 | .00 | .00 | .00 | .00 | .00 | .00 | .00 | .58 | .13 | .00 | .00 |
| G13 | .00 | .15 | .00 | .00 | .00 | .00 | .00 | .00 | .00 | .00 | .00 | .00 | .00 | .00 | .00 | .00 | .00 | .25 | .52 | .20 | .00 |
| G14 | .00 | .09 | .00 | .00 | .00 | .00 | .00 | .00 | .00 | .00 | .00 | .00 | .00 | .00 | .00 | .11 | .00 | .00 | .00 | .47 | .00 |
| G15 | .00 | .11 | .22 | .00 | .00 | .00 | .00 | .00 | .00 | .00 | .00 | .00 | .00 | .00 | .00 | .00 | .00 | .00 | .00 | .00 | .57 |

Abbreviations: P1, delusions; P2, conceptual disorganization; P3, hallucinatory behavior; P6, suspiciousness/persecution; P7, hostility; N1, blunted affect; N2, emotional withdrawal; N3, poor rapport; N4, social withdrawal; N5, difficulty in abstract thinking; N6, lack of spontaneity and flow in conversation; N7, stereotyped thinking; G5, mannerisms and posturing; G7, motor retardation; G8, uncooperativeness; G9, unusual thought content; G10, disorientation; G11, poor attention; G13, disturbance of volition; G14, poor impulse control; G15, preoccupation.

**Table S10.** Complete Edge Weight Matrix for Symptom Interactions in Non-Remitters from Baseline to Week 2 (T0→T1)

|  | **P1** | **P2** | **P3** | **P6** | **P7** | **N1** | **N2** | **N3** | **N4** | **N5** | **N6** | **N7** | **G5** | **G7** | **G8** | **G9** | **G10** | **G11** | **G13** | **G14** | **G15** |
| --- | --- | --- | --- | --- | --- | --- | --- | --- | --- | --- | --- | --- | --- | --- | --- | --- | --- | --- | --- | --- | --- |
| P1 | .54 | .00 | .00 | .18 | .00 | .00 | .00 | .00 | .00 | .00 | .00 | .00 | .00 | .00 | .00 | .18 | .00 | .00 | .00 | .00 | .00 |
| P2 | .00 | .49 | .00 | .00 | .00 | .00 | .00 | .00 | .00 | .00 | .00 | .00 | .00 | .00 | .00 | .00 | .00 | .00 | .00 | .00 | .00 |
| P3 | .00 | .00 | .72 | .12 | .00 | .00 | .00 | .00 | .00 | .13 | .00 | -.16 | .00 | .00 | .00 | .00 | .00 | .00 | .00 | .00 | .00 |
| P6 | .00 | .00 | .00 | .53 | .00 | .15 | .00 | .00 | .00 | .00 | .00 | .00 | .00 | .00 | .00 | .00 | .00 | .00 | .00 | -.14 | .00 |
| P7 | .00 | .00 | .00 | .00 | .58 | .00 | .00 | .00 | .14 | .00 | .00 | .00 | .00 | .00 | .00 | .00 | .00 | .00 | .00 | .16 | .00 |
| N1 | .00 | .00 | .00 | .00 | .00 | .60 | .00 | .13 | .00 | .00 | .00 | .00 | .00 | .00 | .00 | .00 | .00 | .00 | .00 | .00 | .00 |
| N2 | .00 | .00 | .00 | .00 | .00 | .17 | .59 | .17 | .00 | .00 | .00 | .00 | .22 | .00 | .00 | .00 | .00 | .00 | .00 | .00 | .16 |
| N3 | .00 | .00 | .00 | .00 | .00 | .00 | .00 | .52 | .00 | .00 | .00 | .00 | .00 | .00 | .00 | .00 | .00 | .00 | .00 | .00 | .00 |
| N4 | .00 | .00 | .00 | .00 | .00 | .00 | .00 | .00 | .53 | .00 | .00 | .00 | .00 | .00 | .14 | .00 | .19 | .00 | .00 | .00 | .00 |
| N5 | .00 | .11 | .00 | .00 | .00 | .00 | .00 | .00 | .00 | .48 | .00 | .00 | .00 | .00 | .00 | .00 | .00 | .00 | .00 | .00 | .00 |
| N6 | .00 | .00 | .00 | .00 | .00 | .00 | .00 | .00 | .00 | .23 | .46 | .00 | .00 | .00 | .00 | .00 | .00 | .00 | .00 | .00 | .00 |
| N7 | .00 | .00 | .00 | .00 | .00 | .00 | .00 | .00 | .00 | .00 | .00 | .52 | .00 | .00 | .00 | .00 | .00 | .00 | .00 | .00 | .00 |
| G5 | .00 | .00 | .00 | .13 | .00 | .00 | .00 | .00 | .00 | .00 | .00 | .00 | .42 | .00 | .00 | .00 | .00 | .00 | .00 | .00 | .00 |
| G7 | .00 | .00 | -.20 | .00 | .00 | .00 | .00 | .00 | .00 | .00 | .00 | .00 | .00 | .48 | .00 | .00 | .00 | .00 | .00 | -.18 | .00 |
| G8 | .00 | .00 | .00 | .00 | .00 | .00 | .00 | .00 | .00 | .00 | .00 | .00 | .00 | .00 | .54 | .00 | .00 | .00 | .00 | .00 | .00 |
| G9 | .17 | .00 | .00 | .00 | .00 | .00 | .00 | .00 | .00 | .00 | .00 | .00 | .00 | .00 | .00 | .48 | .00 | .00 | .00 | .21 | .00 |
| G10 | .00 | .00 | .00 | .00 | .00 | .00 | .00 | .00 | .00 | .00 | .00 | .00 | .00 | .00 | .14 | .00 | .40 | .00 | .00 | .00 | .00 |
| G11 | .00 | .00 | .00 | .00 | .00 | .00 | .00 | .00 | .00 | .00 | .00 | .00 | .00 | .00 | .00 | .00 | .00 | .48 | .00 | .00 | .00 |
| G13 | .00 | .00 | .00 | .00 | .00 | .00 | .00 | .00 | .00 | .00 | .00 | .00 | .00 | .00 | .00 | .00 | -.29 | .00 | .40 | .00 | .00 |
| G14 | .00 | .00 | .00 | .00 | .00 | .00 | .00 | .00 | .00 | .00 | .00 | .00 | -.12 | .00 | .00 | .00 | .00 | .12 | .00 | .42 | .00 |
| G15 | .00 | .00 | .00 | .00 | .00 | .00 | .00 | .00 | .00 | .00 | .00 | .00 | .00 | .00 | .00 | .00 | .00 | .23 | .00 | .00 | .47 |

Abbreviations: P1, delusions; P2, conceptual disorganization; P3, hallucinatory behavior; P6, suspiciousness/persecution; P7, hostility; N1, blunted affect; N2, emotional withdrawal; N3, poor rapport; N4, social withdrawal; N5, difficulty in abstract thinking; N6, lack of spontaneity and flow in conversation; N7, stereotyped thinking; G5, mannerisms and posturing; G7, motor retardation; G8, uncooperativeness; G9, unusual thought content; G10, disorientation; G11, poor attention; G13, disturbance of volition; G14, poor impulse control; G15, preoccupation.

**Table S11.** Complete Edge Weight Matrix for Symptom Interactions in Non-Remitters from Week 2 to Week 4 (T1→T2)

|  | **P1** | **P2** | **P3** | **P6** | **P7** | **N1** | **N2** | **N3** | **N4** | **N5** | **N6** | **N7** | **G5** | **G7** | **G8** | **G9** | **G10** | **G11** | **G13** | **G14** | **G15** |
| --- | --- | --- | --- | --- | --- | --- | --- | --- | --- | --- | --- | --- | --- | --- | --- | --- | --- | --- | --- | --- | --- |
| P1 | .70 | .00 | .00 | .00 | .00 | .00 | -.16 | .00 | .00 | .00 | -.15 | .00 | -.19 | .00 | .00 | .00 | .00 | .00 | .00 | .00 | .00 |
| P2 | .00 | .61 | .00 | -.18 | .00 | .00 | .00 | -.14 | .00 | .00 | .00 | .00 | .00 | .00 | .00 | .00 | .00 | .00 | .00 | .00 | .00 |
| P3 | .00 | .00 | .73 | .00 | .00 | .00 | .00 | .00 | -.11 | .00 | .00 | .00 | .00 | .00 | .00 | .00 | .00 | .00 | .00 | .00 | .00 |
| P6 | .00 | .00 | .00 | .65 | .00 | .00 | .00 | -.18 | .00 | .00 | .00 | .00 | .00 | -.21 | .00 | .00 | .00 | .00 | .00 | .00 | .00 |
| P7 | .00 | .00 | .00 | .00 | .43 | .00 | .00 | .00 | .00 | .00 | .00 | .00 | .00 | .00 | .00 | .00 | .00 | .14 | .00 | .00 | .00 |
| N1 | .00 | .00 | .00 | .00 | .00 | .54 | .18 | .00 | .00 | .00 | .00 | .00 | .00 | .22 | .00 | .00 | .00 | .00 | .00 | .00 | .00 |
| N2 | .00 | .00 | .00 | .00 | .00 | .00 | .40 | .00 | .22 | .00 | .00 | .00 | .00 | -.15 | .00 | .00 | .00 | .15 | .00 | .00 | .00 |
| N3 | .00 | .00 | .00 | .00 | .00 | .19 | .00 | .42 | .00 | .00 | .24 | .00 | .00 | .24 | .00 | .00 | .00 | .00 | .00 | .00 | .00 |
| N4 | .00 | .00 | .00 | .00 | .00 | .00 | .00 | .00 | .48 | .00 | .00 | .00 | .00 | .00 | .00 | .00 | .00 | .00 | .00 | .00 | .00 |
| N5 | .00 | .00 | .00 | .15 | .00 | .00 | .00 | .00 | .00 | .50 | .00 | .00 | .00 | .00 | .00 | .00 | .27 | .00 | .00 | .00 | .00 |
| N6 | -.19 | .00 | .00 | .00 | .00 | .00 | .00 | .20 | .00 | .00 | .53 | .00 | .00 | .00 | .00 | .00 | .00 | .00 | .00 | .00 | .00 |
| N7 | .00 | .17 | .00 | .00 | .00 | .14 | .00 | .00 | .00 | .16 | .15 | .69 | .00 | .00 | .15 | .00 | .00 | .00 | .17 | .00 | .00 |
| G5 | .00 | .00 | .00 | .00 | .00 | .00 | .00 | .00 | .00 | .00 | .00 | .00 | .56 | .00 | .00 | -.12 | .00 | .00 | .00 | .00 | .00 |
| G7 | .00 | .00 | .00 | .00 | .00 | .00 | .00 | .00 | .00 | .00 | .00 | .00 | .00 | .41 | .00 | .00 | .00 | .13 | .00 | .00 | .00 |
| G8 | .00 | .00 | .00 | .00 | .00 | .00 | .00 | .13 | .00 | .00 | .00 | .00 | .00 | .00 | .54 | .00 | .00 | .00 | .00 | .00 | .00 |
| G9 | .00 | .00 | .00 | .00 | .00 | .00 | .00 | .00 | .00 | .00 | .00 | .00 | .00 | -.13 | -.13 | .58 | .00 | .00 | .00 | .00 | .00 |
| G10 | .00 | .00 | .00 | .13 | .00 | .00 | .00 | .00 | .17 | .00 | .00 | .00 | -.15 | .00 | .00 | .00 | .48 | .00 | .00 | .00 | .00 |
| G11 | .00 | .00 | .00 | .16 | .00 | .00 | .00 | .00 | .00 | .00 | .00 | .00 | .00 | .00 | .00 | .19 | .00 | .33 | .00 | .00 | .00 |
| G13 | .00 | .00 | .00 | .00 | .00 | .00 | .00 | .00 | .00 | .00 | .00 | .00 | .00 | .00 | .00 | .00 | .00 | .21 | .48 | .00 | .00 |
| G14 | .00 | .00 | .00 | .00 | .40 | .00 | .00 | .00 | .11 | .00 | .00 | .00 | .00 | .00 | .00 | .00 | .00 | .00 | .00 | .65 | .00 |
| G15 | .18 | .00 | .00 | .00 | .00 | .00 | .23 | .00 | .00 | .00 | .00 | .00 | .22 | .00 | .00 | .00 | .00 | .00 | .00 | .00 | .58 |

Abbreviations: P1, delusions; P2, conceptual disorganization; P3, hallucinatory behavior; P6, suspiciousness/persecution; P7, hostility; N1, blunted affect; N2, emotional withdrawal; N3, poor rapport; N4, social withdrawal; N5, difficulty in abstract thinking; N6, lack of spontaneity and flow in conversation; N7, stereotyped thinking; G5, mannerisms and posturing; G7, motor retardation; G8, uncooperativeness; G9, unusual thought content; G10, disorientation; G11, poor attention; G13, disturbance of volition; G14, poor impulse control; G15, preoccupation.

**Table S12.** Complete Edge Weight Matrix for Symptom Interactions in Full Sample from Baseline to Week 2 (T0→T1)

|  | **P1** | **P2** | **P3** | **P6** | **P7** | **N1** | **N2** | **N3** | **N4** | **N5** | **N6** | **N7** | **G5** | **G7** | **G8** | **G9** | **G10** | **G11** | **G13** | **G14** | **G15** |
| --- | --- | --- | --- | --- | --- | --- | --- | --- | --- | --- | --- | --- | --- | --- | --- | --- | --- | --- | --- | --- | --- |
| P1 | .41 | .00 | .00 | .10 | .00 | .00 | .00 | .00 | .00 | .00 | .00 | .00 | .00 | .00 | .00 | .10 | .00 | .00 | .00 | .00 | .00 |
| P2 | .00 | .49 | .00 | .00 | .00 | .00 | .00 | .00 | -.07 | .00 | .00 | .00 | .00 | .00 | .00 | .00 | .00 | .00 | .00 | .00 | .00 |
| P3 | .00 | .00 | .59 | .11 | .00 | .00 | .00 | .00 | .00 | .09 | .00 | .00 | .00 | .00 | .00 | .00 | .00 | .00 | .00 | .00 | .00 |
| P6 | .12 | .00 | .00 | .45 | .00 | .00 | .00 | .00 | .00 | .00 | .00 | .00 | .00 | .00 | .00 | .00 | .13 | .00 | .00 | -.12 | .00 |
| P7 | .00 | .00 | .00 | .00 | .50 | .00 | .00 | .00 | .07 | .07 | .00 | .00 | .00 | .00 | .00 | .00 | .00 | .00 | .00 | .12 | .00 |
| N1 | .00 | .00 | .00 | .00 | .00 | .49 | .11 | .00 | .00 | .00 | .00 | .00 | .00 | .00 | .00 | .00 | .00 | .00 | .00 | .00 | .00 |
| N2 | .00 | .00 | .00 | .00 | -.12 | .12 | .45 | .14 | .24 | .00 | .00 | .00 | .18 | .00 | .00 | .00 | .00 | .00 | .00 | .00 | .14 |
| N3 | -.19 | .00 | .00 | .00 | .00 | .00 | .00 | .54 | .00 | .00 | .12 | .00 | .00 | .00 | .00 | -.19 | .00 | .00 | .00 | .00 | .00 |
| N4 | .00 | .00 | .08 | .11 | .22 | .00 | .19 | .00 | .48 | .00 | .00 | .09 | .00 | .00 | .16 | .14 | .16 | .00 | .00 | .13 | .00 |
| N5 | .11 | .10 | .10 | .00 | .00 | .00 | .00 | .00 | .11 | .57 | .00 | .00 | .00 | .00 | .00 | .00 | .10 | .10 | .07 | .00 | .00 |
| N6 | .00 | .00 | .00 | .00 | .00 | .00 | .00 | .00 | .00 | .09 | .44 | .00 | .00 | .00 | .00 | .00 | .00 | .00 | .00 | .00 | .00 |
| N7 | .07 | .08 | .00 | .00 | .00 | .00 | .00 | .00 | .00 | .00 | .00 | .48 | .00 | .10 | .00 | .00 | .00 | .00 | .00 | .00 | .00 |
| G5 | .00 | .00 | .00 | .08 | .00 | .00 | .00 | .08 | .00 | .00 | .00 | .00 | .49 | .00 | .00 | .00 | .00 | .00 | .00 | .00 | .10 |
| G7 | .00 | .00 | -.10 | .00 | .00 | .00 | .00 | .00 | .00 | .00 | .00 | .00 | .00 | .44 | .00 | .00 | .00 | .00 | .00 | -.09 | .00 |
| G8 | .00 | .00 | .00 | .00 | .12 | .00 | .00 | .00 | .00 | .00 | .00 | .00 | .00 | .00 | .56 | .00 | .00 | .00 | .00 | .00 | .00 |
| G9 | .00 | -.07 | .00 | .00 | -.10 | .00 | .00 | .00 | .00 | .00 | .00 | .00 | .00 | .00 | .00 | .38 | -.17 | -.12 | .00 | .00 | .00 |
| G10 | .00 | .00 | .00 | .00 | .00 | .00 | .00 | .00 | .00 | .07 | .00 | .08 | .00 | .00 | .10 | .00 | .44 | .00 | .00 | .00 | .00 |
| G11 | .00 | .00 | .00 | -.12 | .00 | .00 | .00 | .00 | .00 | .00 | .00 | .00 | .00 | .00 | .00 | .00 | .00 | .46 | .00 | .00 | .00 |
| G13 | .14 | .00 | .00 | .00 | .00 | .00 | .00 | .00 | .00 | .00 | .00 | .00 | .00 | .00 | .00 | .00 | -.13 | .00 | .46 | .00 | .00 |
| G14 | .00 | .07 | .00 | .11 | .00 | .00 | .00 | .00 | .00 | .00 | .00 | .00 | .00 | .00 | .00 | .11 | .00 | .00 | .00 | .45 | .00 |
| G15 | .18 | .00 | .00 | .00 | .00 | .00 | .00 | .00 | .00 | .00 | .00 | .09 | .00 | .00 | .00 | .00 | .00 | .17 | .00 | .00 | .50 |

Abbreviations: P1, delusions; P2, conceptual disorganization; P3, hallucinatory behavior; P6, suspiciousness/persecution; P7, hostility; N1, blunted affect; N2, emotional withdrawal; N3, poor rapport; N4, social withdrawal; N5, difficulty in abstract thinking; N6, lack of spontaneity and flow in conversation; N7, stereotyped thinking; G5, mannerisms and posturing; G7, motor retardation; G8, uncooperativeness; G9, unusual thought content; G10, disorientation; G11, poor attention; G13, disturbance of volition; G14, poor impulse control; G15, preoccupation.

**Table S13.** Complete Edge Weight Matrix for Symptom Interactions in Full Sample from Week 2 to Week 4 (T1→T2)

|  | **P1** | **P2** | **P3** | **P6** | **P7** | **N1** | **N2** | **N3** | **N4** | **N5** | **N6** | **N7** | **G5** | **G7** | **G8** | **G9** | **G10** | **G11** | **G13** | **G14** | **G15** |
| --- | --- | --- | --- | --- | --- | --- | --- | --- | --- | --- | --- | --- | --- | --- | --- | --- | --- | --- | --- | --- | --- |
| P1 | .49 | .00 | .00 | .00 | .00 | .00 | .00 | .00 | .10 | .00 | .00 | .00 | .00 | .00 | .00 | .15 | .00 | .00 | .00 | .00 | .00 |
| P2 | .13 | .63 | .00 | .00 | .00 | .00 | .00 | .00 | .00 | .00 | .00 | .00 | .00 | .00 | .00 | .00 | .00 | .00 | .00 | .00 | .00 |
| P3 | .00 | .00 | .66 | .08 | .00 | .00 | .06 | .00 | .00 | .00 | .00 | .00 | .00 | .00 | .00 | .00 | .00 | .00 | .07 | .08 | .00 |
| P6 | .00 | .00 | .00 | .59 | .00 | .00 | .00 | .00 | .00 | .00 | .00 | .00 | .00 | .00 | .00 | .00 | .00 | .00 | .00 | .00 | .07 |
| P7 | .00 | .00 | .00 | .00 | .48 | .00 | .00 | .00 | .00 | .00 | .00 | .00 | .00 | .00 | .15 | .00 | .00 | .00 | .00 | .12 | .00 |
| N1 | .00 | .00 | .00 | .00 | .00 | .55 | .15 | .00 | .00 | .00 | .11 | .00 | .00 | .17 | .00 | .00 | .00 | .00 | .00 | .00 | .00 |
| N2 | .00 | .00 | .00 | .00 | .00 | .13 | .43 | .00 | .17 | .00 | .00 | .00 | .00 | .00 | .00 | .00 | .00 | .00 | .00 | .00 | .00 |
| N3 | .00 | .00 | .00 | .00 | .00 | .12 | .00 | .43 | .00 | .00 | .16 | .00 | .11 | .12 | .00 | .00 | .00 | .00 | .00 | .00 | .00 |
| N4 | .00 | .00 | .00 | .00 | .00 | .00 | .11 | .00 | .43 | .13 | .00 | .00 | .00 | .00 | .10 | .00 | .00 | .00 | .00 | .00 | .00 |
| N5 | .00 | .00 | .00 | .10 | .00 | .00 | .00 | .00 | .00 | .58 | .11 | .00 | .00 | .00 | .00 | .00 | .16 | .00 | .00 | .00 | .00 |
| N6 | -.09 | .00 | .00 | .00 | .00 | .00 | .00 | .14 | .08 | -.10 | .50 | .00 | .00 | .00 | .00 | .00 | .00 | .00 | .00 | .00 | .00 |
| N7 | .00 | .08 | .00 | .00 | .00 | .11 | .00 | .00 | .00 | .10 | .00 | .54 | .00 | .00 | .17 | .00 | .00 | .00 | .10 | .00 | .00 |
| G5 | .00 | .07 | .00 | .00 | .00 | .00 | .00 | .00 | .00 | .00 | .00 | .00 | .56 | .00 | .00 | .00 | .00 | .00 | .00 | .00 | .00 |
| G7 | .07 | .00 | .00 | .00 | .00 | .00 | .00 | .00 | .00 | .00 | .00 | .00 | .00 | .43 | .00 | .00 | .00 | .09 | .00 | .00 | .00 |
| G8 | .00 | .00 | .00 | .00 | .00 | .00 | .07 | .11 | .00 | .00 | .00 | .00 | .00 | .00 | .46 | .00 | .00 | .00 | .00 | .00 | .00 |
| G9 | .09 | .00 | .00 | .00 | .00 | .00 | .00 | .00 | .00 | .00 | .00 | .00 | .00 | .00 | .00 | .52 | .00 | .00 | .00 | .00 | .00 |
| G10 | .00 | .00 | .00 | .00 | .00 | .00 | .00 | .00 | .00 | .00 | .00 | .00 | -.15 | .00 | .00 | .00 | .53 | .00 | .00 | .00 | .00 |
| G11 | -.08 | .00 | .00 | .00 | .00 | .00 | .00 | .00 | .00 | .00 | .00 | .00 | .00 | .00 | .00 | .00 | .00 | .52 | .15 | .00 | .09 |
| G13 | .08 | .00 | .00 | .00 | .00 | .00 | .00 | .00 | .00 | -.12 | .00 | .00 | .00 | .00 | .00 | .00 | .00 | .13 | .44 | .00 | .00 |
| G14 | .00 | .00 | .00 | .00 | .11 | .00 | .00 | .00 | .00 | .00 | .00 | .00 | .00 | .00 | .00 | .00 | .00 | .00 | .00 | .48 | .00 |
| G15 | .06 | .00 | .00 | .00 | .00 | .00 | .12 | .00 | .08 | .00 | .00 | .09 | .22 | .00 | -.13 | .00 | .00 | .00 | .00 | .00 | .55 |

Abbreviations: P1, delusions; P2, conceptual disorganization; P3, hallucinatory behavior; P6, suspiciousness/persecution; P7, hostility; N1, blunted affect; N2, emotional withdrawal; N3, poor rapport; N4, social withdrawal; N5, difficulty in abstract thinking; N6, lack of spontaneity and flow in conversation; N7, stereotyped thinking; G5, mannerisms and posturing; G7, motor retardation; G8, uncooperativeness; G9, unusual thought content; G10, disorientation; G11, poor attention; G13, disturbance of volition; G14, poor impulse control; G15, preoccupation

1. **Supplementary Figures**

**Figure S1.** Baseline Networks of Non-Remitters and Matched Remitters

**B**

**A**

|  | **P1** | Delusions |
| --- | --- | --- |
|  | **P2** | Conceptual disorganization |
|  | **P3** | Hallucinations |
|  | **P6** | Suspiciousness/persecution |
|  | **P7** | Hostility |
|  | **N1** | Blunted affect |
|  | **N2** | Emotional withdrawal |
|  | **N3** | Poor rapport |
|  | **N4** | Social withdrawal |
|  | **N5** | Difficulty in abstract thinking |
|  | **N6** | Lack of spontaneity and flow of conversation |
|  | **N7** | Stereotyped thinking |
|  | **G5** | Mannerisms and posturing |
|  | **G7** | Motor retardation |
|  | **G8** | Uncooperativeness |
|  | **G9** | Unusual thought content |
|  | **G10** | Disorientation |
|  | **G11** | Poor attention |
|  | **G13** | Disturbance of volition |
|  | **G14** | Poor impulse control |
|  | **G15** | Preoccupation |

**C**

|  | Positive |  | Negative |  | Excited/Aggressive |  | Cognitive/Disorganized |
| --- | --- | --- | --- | --- | --- | --- | --- |

Baseline networks and Expected Influence centrality of non-remitters and matched remitters. A, Network of remitters (N = 196). B, Network of non-remitters (N = 196). C, Expected Influence and strength centrality for remitters and non-remitters (z-scored). In the network, blue lines represent positive associations, while red dashed lines represent negative associations. Edge thickness indicates the strength of the association, with thicker edges reflecting stronger associations. The maximum edge value for both networks was standardized to 0.54, which corresponds to the highest observed association.

**Figure S2.** Bootstrapped Confidence Intervals of Estimated Edge Weights

**A                                                                                  B**

The red line indicates the sample values and the grey area indicates the bootstrapped confidence intervals. Each horizontal line represents one edge of the network ordered by edge-weights. A, Remitters. B, Non-remitters.

**Figure S3.** The Stability of Expected Influence Centrality Using Case-Dropping Bootstrap


**A                                                                            B**

Mean correlations between Expected Influence centrality of original sample and sub samples with different degrees of persons dropped. Lines reflect means and areas around the lines reflect 95% confidence intervals. A, Remitters. B, Non-remitters.

**Figure S4.** Item-Level Symptom Severity Over Time for Remitters and Non-Remitters

**A**

**B**

A, Remitters. B, Non-remitters. Abbreviations: P1, delusions; P2, conceptual disorganization; P3, hallucinatory behavior; P6, suspiciousness/persecution; P7, hostility; N1, blunted affect; N2, emotional withdrawal; N3, poor rapport; N4, social withdrawal; N5, difficulty in abstract thinking; N6, lack of spontaneity and flow in conversation; N7, stereotyped thinking; G5, mannerisms and posturing; G7, motor retardation; G8, uncooperativeness; G9, unusual thought content; G10, disorientation; G11, poor attention; G13, disturbance of volition; G14, poor impulse control; G15, preoccupation.

**Figure S5**. Cross-Lagged Panel Networks for Remitters and Non-Remitters Including Auto-Regressive Edges

**A**

**B**

**D**

**C**

**A**

| **Positive** | **Negative** | | **Excited/Aggressive** | | **Cognitive/Disorganized** |
| --- | --- | --- | --- | --- | --- |
| **P1.** Delusions | | **N3.** Poor rapport | | **G8.** Uncooperativeness | |
| **P2.** Conceptual disorganization | | **N4.** Social withdrawal | | **G9.** Unusual thought content | |
| **P3.** Hallucinations | | **N5.** Difficulty in abstract thinking | | **G10.** Disorientation | |
| **P6.** Suspiciousness/persecution | | **N6.** Lack of spontaneity and flow of  conversation | | **G11.** Poor attention | |
| **P7.** Hostility | | **N7.** Stereotyped thinking | | **G13.** Disturbance of volition | |
| **N1.** Blunted affect | | **G5.** Mannerisms and posturing | | **G14.** Poor impulse control | |
| **N2.** Emotional withdrawal | | **G7.** Motor retardation | | **G15.** Preoccupation | |

Cross-Lagged Panel Network model plots for T0→T1 Network and T1→T2 Network, including autoregressive edges. A, T0→T1 network of remitters. B, T1→T2 network of remitters. C, T0→T1 network of non-remitters. D, T1→T2 network of non-remitters. The direction of the edges indicates the direction of the cross-lagged coefficients between nodes. Dashed red edges signify negative associations, while blue edges represent positive associations. The saturation and thickness of the edges indicate the strength of the associations. Only significant (*P* <.05) edges are visualized. The maximum edge value for both networks was standardized to 0.73, which corresponds to the highest observed association.

**Figure S6.** Autoregressive Edges for T0→T1 and T1→T2 Networks of Remitters and Non-Remitters

**B**

**A**

| **Positive** | **Negative** | | **Excited/Aggressive** | | **Cognitive/Disorganized** |
| --- | --- | --- | --- | --- | --- |
| **P1.** Delusions | | **N3.** Poor rapport | | **G8.** Uncooperativeness | |
| **P2.** Conceptual disorganization | | **N4.** Social withdrawal | | **G9.** Unusual thought content | |
| **P3.** Hallucinations | | **N5.** Difficulty in abstract thinking | | **G10.** Disorientation | |
| **P6.** Suspiciousness/persecution | | **N6.** Lack of spontaneity and flow of  conversation | | **G11.** Poor attention | |
| **P7.** Hostility | | **N7.** Stereotyped thinking | | **G13.** Disturbance of volition | |
| **N1.** Blunted affect | | **G5.** Mannerisms and posturing | | **G14.** Poor impulse control | |
| **N2.** Emotional withdrawal | | **G7.** Motor retardation | | **G15.** Preoccupation | |

Autoregressive edges for each item in the network. A, T0→T1 network. B, T1→T2 network. Solid black lines represent non-remitters and dashed black lines remitters.

**Figure S7.** Bootstrapped Confidence Intervals of Estimated Edge Weights for the CLPN Models of Remitters and Non-Remitters

**A**

**B**

**D**

**C**

Bootstrapped 95% confidence intervals (CIs) for the estimated edge weights of the remitter and non-remitter networks at T0→T1 and T1→T2. A, Remitters at T0→T1. B, Remitters at T1→T2. C, Non-remitters at T0→T1. D, Non-remitters at T1→T2. Each horizontal line represents a distinct edge from the Cross-Lagged Panel Network model. The gray horizontal bars indicate the 95% CIs for each edge's weight. The red dot on each line represents the sample's estimated edge weight, while the black dot signifies the bootstrap mean of the edge weight. Crucially, this figure only includes edges that were estimated as non-zero by the preceding LASSO model, thereby encompassing both statistically significant and non-significant edges. The larger the gray line the less certain the edge value is. Abbreviations: LASSO, Least Absolute Shrinkage and Selection Operator.

**Figure S8.** Bootstrapped Sampling Distribution


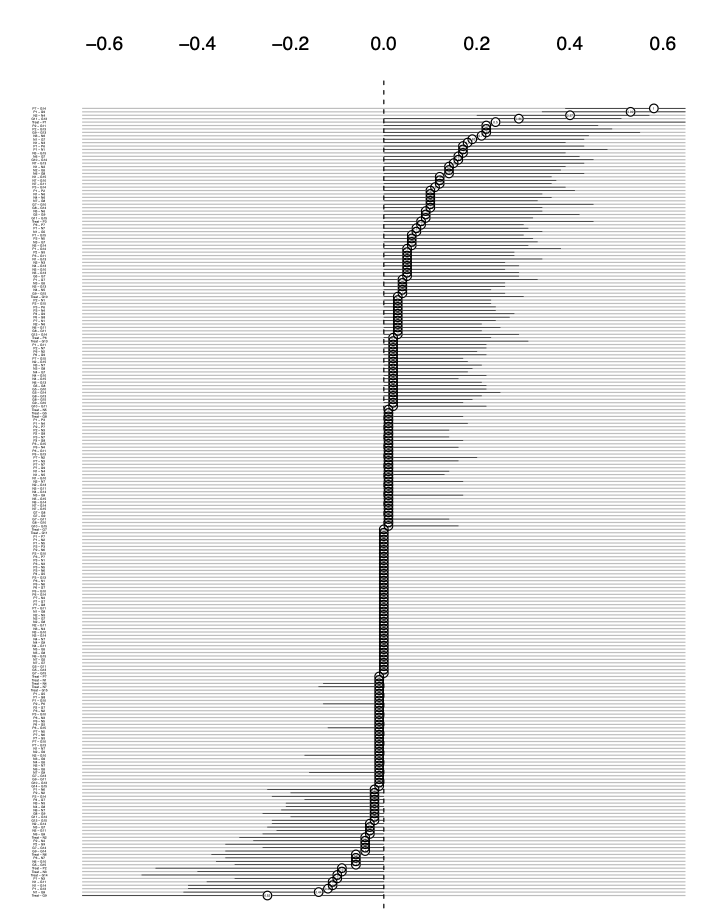


The plot shows the estimated edge weights (black circles) with their corresponding 95% confidence intervals (gray lines). The dashed vertical line at zero indicates no effect. The edge between treatment and N3 (Poor rapport) was present in only 31% of bootstrap samples, indicating low stability of this relationship.

**Figure S9** Item-Level Symptom Severity Over Time for Full Sample

Abbreviations: P1, delusions; P2, conceptual disorganization; P3, hallucinatory behavior; P6, suspiciousness/persecution; P7, hostility; N1, blunted affect; N2, emotional withdrawal; N3, poor rapport; N4, social withdrawal; N5, difficulty in abstract thinking; N6, lack of spontaneity and flow in conversation; N7, stereotyped thinking; G5, mannerisms and posturing; G7, motor retardation; G8, uncooperativeness; G9, unusual thought content; G10, disorientation; G11, poor attention; G13, disturbance of volition; G14, poor impulse control; G15, preoccupation.

**Figure S10.** Cross-Lagged Panel Network Models for Full Sample

**B**

**A**

| **Positive** | **Negative** | | **Excited/Aggressive** | | **Cognitive/Disorganized** |
| --- | --- | --- | --- | --- | --- |
| **P1.** Delusions | | **N3.** Poor rapport | | **G8.** Uncooperativeness | |
| **P2.** Conceptual disorganization | | **N4.** Social withdrawal | | **G9.** Unusual thought content | |
| **P3.** Hallucinations | | **N5.** Difficulty in abstract thinking | | **G10.** Disorientation | |
| **P6.** Suspiciousness/persecution | | **N6.** Lack of spontaneity and flow of  conversation | | **G11.** Poor attention | |
| **P7.** Hostility | | **N7.** Stereotyped thinking | | **G13.** Disturbance of volition | |
| **N1.** Blunted affect | | **G5.** Mannerisms and posturing | | **G14.** Poor impulse control | |
| **N2.** Emotional withdrawal | | **G7.** Motor retardation | | **G15.** Preoccupation | |

Cross-Lagged Panel Network model plots for T0→T1 network and T1→T2 network. A, T0→T1 network. B, T1→T2 network. The direction of the edges indicates the direction of the cross-lagged coefficients between nodes. Dashed red edges signify negative associations, while blue edges represent positive associations. The saturation and thickness of the edges indicate the strength of the associations. Only significant (*P* < .05) edges are visualized. The maximum edge value for both networks was standardized to 0.24, which corresponds to the highest observed association.

**Figure S11.** Cross-Lagged In-and-Out Prediction for Full Sample

**A**

**B**

| **Positive** | **Negative** | | **Excited/Aggressive** | | **Cognitive/Disorganized** |
| --- | --- | --- | --- | --- | --- |
| **P1.** Delusions | | **N3.** Poor rapport | | **G8.** Uncooperativeness | |
| **P2.** Conceptual disorganization | | **N4.** Social withdrawal | | **G9.** Unusual thought content | |
| **P3.** Hallucinations | | **N5.** Difficulty in abstract thinking | | **G10.** Disorientation | |
| **P6.** Suspiciousness/persecution | | **N6.** Lack of spontaneity and flow of  conversation | | **G11.** Poor attention | |
| **P7.** Hostility | | **N7.** Stereotyped thinking | | **G13.** Disturbance of volition | |
| **N1.** Blunted affect | | **G5.** Mannerisms and posturing | | **G14.** Poor impulse control | |
| **N2.** Emotional withdrawal | | **G7.** Motor retardation | | **G15.** Preoccupation | |

In-and-out prediction plots for full sample. A, In-prediction for T0→T1 (left) and T1→T2 (right). B, Out-prediction for T0→T1 (left) and T1→T2 (right). The x-axis represents the explained variance.

**Figure S12.** Cross-Lagged Panel Networks for Full Sample Including Autoregressive Edges

**A                                                                                  B**

| **Positive** | **Negative** | | **Excited/Aggressive** | | **Cognitive/Disorganized** |
| --- | --- | --- | --- | --- | --- |
| **P1.** Delusions | | **N3.** Poor rapport | | **G8.** Uncooperativeness | |
| **P2.** Conceptual disorganization | | **N4.** Social withdrawal | | **G9.** Unusual thought content | |
| **P3.** Hallucinations | | **N5.** Difficulty in abstract thinking | | **G10.** Disorientation | |
| **P6.** Suspiciousness/persecution | | **N6.** Lack of spontaneity and flow of  conversation | | **G11.** Poor attention | |
| **P7.** Hostility | | **N7.** Stereotyped thinking | | **G13.** Disturbance of volition | |
| **N1.** Blunted affect | | **G5.** Mannerisms and posturing | | **G14.** Poor impulse control | |
| **N2.** Emotional withdrawal | | **G7.** Motor retardation | | **G15.** Preoccupation | |

Cross-Lagged Panel Network model plots for T0→T1 Network and T1→T2 Network, including auto-regressive edges. A, T0→T1 network. B, T1→T2 network. The direction of the edges indicates the direction of the cross-lagged coefficients between nodes. Dashed red edges signify negative associations, while blue edges represent positive associations. The saturation and thickness of the edges indicate the strength of the associations. Only significant (*P* < .05) edges are visualized. The maximum edge value for both networks was standardized to 0.66, which corresponds to the highest observed association.

**Figure S13.** Autoregressive Edges for T0→T1 and T1→T2 Networks of the Full Sample

| **Positive** | **Negative** | | **Excited/Aggressive** | | **Cognitive/Disorganized** |
| --- | --- | --- | --- | --- | --- |
| **P1.** Delusions | | **N3.** Poor rapport | | **G8.** Uncooperativeness | |
| **P2.** Conceptual disorganization | | **N4.** Social withdrawal | | **G9.** Unusual thought content | |
| **P3.** Hallucinations | | **N5.** Difficulty in abstract thinking | | **G10.** Disorientation | |
| **P6.** Suspiciousness/persecution | | **N6.** Lack of spontaneity and flow of  conversation | | **G11.** Poor attention | |
| **P7.** Hostility | | **N7.** Stereotyped thinking | | **G13.** Disturbance of volition | |
| **N1.** Blunted affect | | **G5.** Mannerisms and posturing | | **G14.** Poor impulse control | |
| **N2.** Emotional withdrawal | | **G7.** Motor retardation | | **G15.** Preoccupation | |

Autoregressive edges for each item in the full sample network. Solid black lines represent T0→T1 and dashed black lines T1→T2.

**Figure S14.** Bootstrapped Confidence Intervals of Estimated Edge Weights for the CLPN Models of the Full Sample

**A**

**B**

Bootstrapped 95% confidence intervals (CIs) for the estimated edge weights of the full sample networks at T0→T1 and T1→T2. A, T0→T1. B, T1→T2. Each horizontal line represents a distinct edge from the Cross-Lagged Panel Network model. The gray horizontal bars indicate the 95% CIs for each edge's weight. The red dot on each line represents the sample's estimated edge weight, while the black dot signifies the bootstrap mean of the edge weight. Crucially, this figure only includes edges that were estimated as non-zero by the preceding LASSO model, thereby encompassing both statistically significant and non-significant edges. The larger the gray line the less certain the edge value is. Abbreviations: LASSO, Least Absolute Shrinkage and Selection Operator.

1. **References**
2. Rubin M, Bicki A, Papini S, et al. Distinct trajectories of depression symptoms in early and middle adolescence: Preliminary evidence from longitudinal network analysis. J Psychiatr Res. 2021;142:198-203.
3. Wang S, Chong ZY, Zhang C, Xu W. Longitudinal associations between anxiety and depressive symptoms in adolescence, early adulthood, and old age: Cross-lagged panel network analyses. Depress Anxiety. 2024;2024(1):6205475.
4. Zhao H, Zhou A. Longitudinal relations between non-suicidal self-injury and both depression and anxiety among senior high school adolescents: A cross-lagged panel network analysis. PeerJ. 2024;12:e18134.
5. Zhao Y, Liang K, Qu D, et al. Unraveling depressive symptom networks: A three-year longitudinal study among Chinese junior high school adolescents. J Res Adolesc. 2025;35(1):e13040.
6. Jaccard P. Étude comparative de la distribution florale dans une portion des Alpes et des Jura. Bull Soc Vaudoise Sci Nat. 1901;37:547-579.
7. Costa LDF. Further generalizations of the Jaccard index. arXiv preprint arXiv:2110.09619. Published 2021.
8. Dal Santo F, García-Portilla MP, Fernández-Egea E, et al. The dimensional structure of the Positive and Negative Syndrome Scale in first-episode schizophrenia spectrum disorders: an exploratory graph analysis from the OPTiMiSE trial. Schizophrenia. 2024;10(1):81. doi:10.1186/s41537-024-00179-3.
